# Na^+^/H^+^-exchanger 1 Enhances Antitumor Activity of Engineered NK-92 Natural Killer Cells

**DOI:** 10.1158/2767-9764.CRC-22-0270

**Published:** 2022-08-22

**Authors:** Yao-Yu Gong, Hongguang Shao, Yu Li, Patricia Brafford, Zachary E. Stine, Jing Sun, Dean W. Felsher, Jordan S. Orange, Steven M. Albelda, Chi V. Dang

**Affiliations:** 1 Cell and Molecular Biology Graduate Group, Perelman School of Medicine, University of Pennsylvania, Philadelphia, Pennsylvania.; 2 The Wistar Institute, Philadelphia, Pennsylvania.; 3 Department of Pediatrics, Columbia University Medical Center, New York, New York.; 4 Department of Medicine, Perelman School of Medicine, University of Pennsylvania, Philadelphia, Pennsylvania.; 5 Division of Oncology, Department of Medicine, Stanford University School of Medicine, Stanford, California.; 6 Ludwig Institute for Cancer Research, New York, New York.

## Abstract

Adoptive cell transfer (ACT) immunotherapy has remarkable efficacy against some hematologic malignancies. However, its efficacy in solid tumors is limited by the adverse tumor microenvironment (TME) conditions, most notably that acidity inhibits T and natural killer (NK) cell mTOR complex 1 (mTORC1) activity and impairs cytotoxicity. In several reported studies, systemic buffering of tumor acidity enhanced the efficacy of immune checkpoint inhibitors. Paradoxically, we found in a *MYC*–driven hepatocellular carcinoma model that systemic buffering increased tumor mTORC1 activity, negating inhibition of tumor growth by anti-PD1 treatment. Therefore, in this proof-of-concept study, we tested the metabolic engineering of immune effector cells to mitigate the inhibitory effect of tumor acidity while avoiding side effects associated with systemic buffering. We first overexpressed an activated RHEB in the human NK cell line NK-92, thereby rescuing acid-blunted mTORC1 activity and enhancing cytolytic activity. Then, to directly mitigate the effect of acidity, we ectopically expressed acid extruder proteins. Whereas ectopic expression of carbonic anhydrase IX (CA9) moderately increased mTORC1 activity, it did not enhance effector function. In contrast, overexpressing a constitutively active Na^+^/H^+^-exchanger 1 (NHE1; *SLC9A1*) in NK-92 did not elevate mTORC1 but enhanced degranulation, target engagement, *in vitro* cytotoxicity, and *in vivo* antitumor activity. Our findings suggest the feasibility of overcoming the inhibitory effect of the TME by metabolically engineering immune effector cells, which can enhance ACT for better efficacy against solid tumors.

**Significance:**

This study demonstrates the feasibility of metabolic engineering immune effector cells to overcome inhibition in the TME, an approach that could enhance the efficacy of adoptive transfer immunotherapy.

## Introduction

The advancement of cancer immunotherapy has revolutionized the standard of care for various malignancies, including solid tumors like malignant melanoma and lung cancer (1). The unprecedented results obtained by immunotherapies, such as immune checkpoint blockade (ICB), highlight the potential of empowering the patients’ own immune systems to treat cancer.

More recently, adoptive cell transfer (ACT) has emerged as a promising strategy for immunotherapy. ACT involves the autologous or allogeneic transfer of tumor-infiltrating lymphocytes or genetically engineered T cells expressing T-cell receptors or chimeric antigen receptors (CAR) targeting specific tumor antigens (2). Additional attempts have been made to use natural killer (NK) cells or CAR-modified NK cells for ACT, leveraging their potentially better safety profiles than T cells (3). However, although ACT has demonstrated remarkable efficacy in patients with hematologic malignancies, leading to long-lasting response or even complete remission in some cases, it has resulted in a low overall response rate of 9% among clinical trials for solid tumors (4). As a result, the clinical implementation of ACT to treat solid tumors is still in its infancy.

A significant hurdle for ACT in solid tumors is the immunosuppressive tumor microenvironment (TME). The TME of solid tumors is not only marked by the presence of immunosuppressive cells, ligands, and cytokines but also by environmental adversities such as nutrient depletion, hypoxia, and acidosis (5). Compared with healthy tissues, the extracellular pH (pH_e_) of tumors is often acidic. For example, the pH_e_ of malignant melanoma ranges from 6.4 to 7.3, whereas the pH_e_ of the normal dermis is 7.2 to 7.6 (6). The acidic pH_e_ is mainly a result of aberrant stromal or tumor cell metabolism that favors glycolysis driven by hypoxia or the activity of oncogenes such as *MYC* (7). Glycolysis produces a net of two protons for every glucose consumed. Furthermore, glucose is converted to lactic acid, an acidic byproduct released to the TME by monocarboxylate transporters (MCT) 1 and 4 (8). However, an acidic TME may not be limited to glycolytic tumors, as tumors lacking key glycolysis enzymes can still acidify their pH_e_ by hydrating carbon dioxide (CO_2_) produced by oxidative phosphorylation (9). While tumor cells acidify the TME, they typically maintain an alkaline intracellular pH (pH_i_) with the help of acid extruder proteins such as MCT1 and MCT4 (8). Besides the MCTs, tumor cells also express carbonic anhydrases (CA) for the hydration of CO_2_, various proton exchangers and pumps for the direct export of proton, and bicarbonate transporters for the uptake of bicarbonates to neutralize intracellular acids (8). The expression of these acid extruder proteins in tumor cells is often induced by metabolic conditions cooccurring with acidosis, such as hypoxia. For example, the plasma membrane CA9 is upregulated by hypoxia-inducible factor (HIF) 1α to counteract intracellular acidosis (10). Hypoxia also upregulates MCT4 in a HIF1α-dependent mechanism (11).

Although tumor cells are protected from acidosis by acid extruder proteins, the infiltrating immune effector cells in the TME are vulnerable to its acidity due to their paucity of such proteins (12). This vulnerability has important implications as a growing body of research has suggested that immune effector cells, including CD8^+^ T cells and NK cells, lose their antitumor cytotoxicity in acidic environments. For example, primary human CD8^+^ T cells lose cytokine production and antitumor cytotoxicity in acidic culture media (13). Similarly, primary mouse NK cells also show diminished cytolytic activity in acidic environments (14). In both cases, the presence of lactate is not necessary to inhibit the cytotoxicity of the effector cells, as acidification caused by inorganic acids, such as hydrochloric acid, is sufficient. Such *in vitro* findings are corroborated by *in vivo* experiments where systemic buffering by oral sodium bicarbonate (NaHCO_3_) reinvigorates immune effector cells, including NK cells and T cells, in mice (14, 15). Moreover, systemic buffering can boost response to immunotherapies, including ICB and ACT with T cells, leading to complete remission of xenograft melanoma in some mice (15). Similarly, others have treated mice systemically with the proton pump inhibitor (PPI) esomeprazole targeting H^+^/K^+^-ATPase and saw enhanced efficacy of ACT with T cells (16).

Despite the successes of these systemic strategies to tackle tumor acidosis, it remains possible that systemic buffering may unexpectedly benefit tumor cells by relieving the negative impact of acidosis on them. Indeed, clinical trials of immune checkpoint inhibitors with concomitant PPIs reported mixed results, with an overall deleterious effect evidenced by increased disease progression and death (17). In the study reported here, we found that systemic buffering by oral NaHCO_3_ paradoxically negated the effect of anti-PD1, an immune checkpoint inhibitor, in an inducible *MYC*–driven mouse hepatocellular carcinoma (HCC) model (18). Therefore, we focused on metabolically engineering immune effector cells as an alternative approach to specifically mitigate the inhibitory effect of the acidic TME on immune effector cells. Using the human NK cell line NK-92 as a model of immune effector cells, we first demonstrated the feasibility of our metabolic engineering approach by overexpressing RHEB, which rescued the acid-blunted mTORC1 activity (19) and enhanced the cytolytic activity of the cells. We then sought to generate acid–resistant NK-92 cells by overexpressing acid extruder proteins CA9 or Na^+^/H^+^-exchanger 1 (NHE1). We found that NHE1 enhanced the antitumor activity of NK-92 cells *in vitro* and *in vivo* by enhancing their degranulation. These findings demonstrate the feasibility of engineering immune effector cells to overcome adverse immunosuppressive metabolic conditions in the TME and potentially enhance the efficacy of ACT against solid tumors.

## Materials and Methods

### Cell Culture and Media

NK-92 cells (20) and NK-92MI cells (21) were purchased from the ATCC (CRL-2407, RRID:CVCL_2142, and CRL-2408, RRID:CVCL_3755, respectively). Cells were maintained in RPMI1640 with 25 mmol/L HEPES (Corning, 10-041-CV) supplemented with 10% FBS (GeminiBio, 900-108), sodium pyruvate (1 mmol/L), l-glutamine (2 mmol/L), MEM Non-Essential Amino Acids (1 ×) (Gibco, 11140-050), and 2-mercaptoethanol (2 ×) (Gibco, 21985-023). In addition, for NK-92 cells, 300 IU/mL of recombinant human IL2 (STEMCELL Technologies, 78036) was added prior to use. Human melanoma cell lines WM3629 (RRID:CVCL_C275), WM4237, and WM1727A (22) were gifts from Dr. Meenhard Herlyn at the Wistar Institute. K562 cells were purchased from the ATCC (CCL-243). Both melanoma cells and K562 cells were maintained in RPMI1640 with 25 mmol/L HEPES supplemented with 5% FBS. All cell lines were routinely authenticated by short tandem repeat profiling and tested for *Mycoplasma* contamination.

pH-controlled culture media for NK-92 cells were prepared following a previously reported method (23). Briefly, the media stock was prepared using complete NK-92 culture media plus 20 mmol/L HEPES and 20 mmol/L PIPES and adjusted to the desired pH using concentrated NaOH or HCl solutions. NaHCO_3_ was added at 18, 11.4, 7, 4.3, 2.7, and 0.8 mmol/L for pH 7.4, 7.2, 7.0, 6.8, 6.6, and 6.3 media, respectively, as determined by a titration process described in the cited work. The varying amount of NaHCO_3_ ensures that the media maintain their desired pH in incubators with 5% CO_2_.

### Stable Overexpression

The lentiviral vector encoding constitutively active human RHEB was generated by cloning the RHEB^N153T^ cDNA of the plasmid pcDNA3-FLAG-Rheb-N153T (Addgene, 19997, RRID:Addgene_19997), a kind gift from Dr. Fuyuhiko Tamanoi (24), into pHIG2PW, a lentiviral transfer vector allowing the expression of EGFP and puromycin resistance gene alongside the transgene. Lentiviral vectors encoding human CA9 and constitutively active human NHE1 (gene symbol *SLC9A1*) were generated by cloning synthesized human *CA9 cDNA* and codon-optimized human *NHE1 cDNA* fragments (GeneCopoeia; Supplementary Table S1) with mutated histidine clusters (25) into pHIG2PW. The lentiviral vector encoding inactive mutant NHE1 bearing E262I mutation was generated by site-directed mutagenesis of the constitutively active NHE1 vector using Q5 Site-Directed Mutagenesis Kit (New England Biolabs, E0554S) with the following custom primers: 5′-CGTTTTCGGTATCAGTCTTCTCAATG-3′ and 5′-AGTATATGAAGAAGTTCGTTG-3′. The lentiviral vector encoding human SERPINB9 was generated by cloning the SERPINB9 cDNA from a human ORF clone (GenScript, OHu01596) into pHIG2PW.

All lentiviruses were packaged by cotransfecting the transfer plasmids with second-generation packaging and envelope plasmids pMD2.G and psPAX2, gifts of Dr. Didier Trono (Addgene, 12259, and RRID:Addgene_12260). Lentiviruses were purified and concentrated by centrifugation as described before (26) and were used to transduce NK-92 or NK-92MI cells. Briefly, NK-92 or NK-92MI cells were stimulated with 500 IU/mL of recombinant human IL2 (STEMCELL Technologies, 78036) and 50 ng/mL of recombinant human IL21 (R&D Systems, 8879-IL-010) overnight prior to the addition of concentrated lentiviruses. Cells were incubated with lentiviruses for 4 days, followed by puromycin selection (2 μg/mL) for 2 weeks. Finally, stably transduced cells were sorted by EGFP using a cell sorter.

### Animal Studies

All animal experiments were approved by the Institutional Animal Care and Use Committee at the Wistar Institute (protocol numbers 201189 and 201379) and were executed in compliance with institutional guidelines and regulations. All animals were kept in a specific pathogen-free facility and provided drinking water and food *ad libitum*. Littermates were randomized before each experiment.

To study the effect of systemic buffering on the efficacy of checkpoint blockade immunotherapy, LAP/*MYC* transgenic mice, which develop *MYC*–driven neonatal HCC unless treated with doxycycline (18), were obtained from Dr. Dean Felsher at Stanford University (Stanford, *CA*) and bred at Charles River Laboratory. In these animals, the inducible *MYC* allele was integrated into an autosome, and animals were maintained in the homozygous states of the activator LAP (Tet-Off) or *MYC*. Animals (males and females) were bred on demand to generate compound heterozygotes of LAP and *MYC*. Mice were fed with doxycycline-containing (200 mg/kg) chow diet and then shipped at age 6 weeks for experiments. Transgenic *MYC* was activated at 7 weeks after birth by removal of doxycycline chow, and then animals were provided with regular or NaHCO_3_-containing (200 mmol/L) drinking water. To study the effect of systemic buffering on the efficacy of ICB, mice were treated with either 12 mg/kg of GoInVivo purified anti-mouse CD279 (PD1) antibody (BioLegend, 135235) or isotype control antibody (BioLegend, 400564) via intraperitoneal injection at 8 weeks for 4 weeks and then sacrificed. Livers were weighed as surrogate of tumor load. We showed previously that tumor clusters could be seen at necropsy after 3 weeks of *MYC* induction at birth by withdrawing doxycycline from drinking water used by mothers and subsequently by pups (27). Gross increase in abdominal girth was apparent at 7 weeks and by 15 weeks, all animals died.

To test the *in vivo* cytotoxicity of NHE1-expressing NK-92MI cells, we used an adoptive transfer model. Female NSG mice of 9 to 12 weeks of age were obtained from the Animal Facility at the Wistar Institute. WM3629 tumor xenografts were generated by subcutaneously injecting 3 × 10^5^ cells in a 1:1 mixture of PBS and Matrigel Growth Factor Reduced (Corning, 354230) at the flanks of the mice. Once the xenografts reached a volume of 100 mm^3^, 2.5 × 10^6^ NK-92MI cells in PBS were intravenously injected in four doses (one initial dose of 1 × 10^6^ cells, followed by four booster doses of 0.5 × 10^6^ cells) with 3- or 4-day intervals. Untreated control mice were injected with equal volumes of PBS.

### Histology and IHC

At the endpoints of animal experiments, tumors were harvested, fixed in 10% phosphate-buffered formalin, embedded in paraffin, and sliced into sections. Slides were stained with hematoxylin and eosin stain.

To assess mTORC1 activity and apoptosis in tumor samples, IHC for phospho-S6 ribosomal protein (pS6) and cleaved caspase-3 was performed, respectively. Briefly, slides were deparaffinized and treated with Dako Target Retrieval Solution, pH 9 (Agilent, S236784-2), following the manufacturer's protocol. Rabbit anti-pS6 (Ser235/Ser236) primary antibody (Cell Signaling Technology, 2211, 1:400) or rabbit anti-cleaved caspase-3 (Asp175) antibody (Cell Signaling Technology, 9661, RRID:AB_2341188, 1:400) was applied, followed by horseradish peroxidase–conjugated secondary antibody. A solution containing the chromogen 3,3′-diaminobenzidine was used to detect the antibodies, followed by subsequent hematoxylin counterstain.

### Flow Cytometry

To study the effect of Torin 1 or pH_e_ on mTORC1 activity in NK-92 cells, cells were treated with Torin 1 or pH-controlled culture media for 4 hours before being fixed with 4% paraformaldehyde (PFA) in PBS for 20 minutes at room temperature. Cells were washed with PBS before permeabilization using Intracellular Staining Permeabilization Wash Buffer (BioLegend, 421002) according to the manufacturer's directions. Cells were then stained with PE-Cy7–conjugated rabbit anti-pS6 (Ser235/Ser236) antibody (Cell Signaling Technology, 34411) or isotype control antibody (Cell Signaling Technology, 97492) at 1:50 dilution for 20 minutes on ice before analysis.

To assess the effect of trametinib or target cell engagement on ERK activity in NK-92 cells, cells were treated with trametinib for 1 hour or incubated with K562 cells at 1:1 ratio for 0 to 2 hours before being fixed with 4% PFA in PBS for 20 minutes at room temperature. Cells were then permeabilized with ice-cold methanol for 20 minutes with constant, gentle agitation, washed with PBS, and stained with PE-Cy7–conjugated rabbit anti-phospho-p44/42 MAPK (pERK1/2; Thr202/Tyr204) antibody (Cell Signaling Technology, 98168) or appropriate isotype control antibody (Cell Signaling Technology, 97492) at 1:50 dilution for 30 minutes on ice before analysis.

To assess the basal levels of granzyme B or perforin in NK-92 cells, untreated cells were fixed, permeabilized, and stained following the same procedures for pS6 staining. For granzyme B, cells were stained with PerCP-Cy5.5–conjugated mouse anti-granzyme B antibody (BioLegend, 372211, RRID:AB_2728378) or isotype control antibody (BioLegend, 400149) at 1:50 dilution. For perforin, cells were stained with PE-Dazzle 594-conjugated mouse anti-human perforin antibody (BioLegend, 308131, RRID:AB_916156) or corresponding isotype control antibody (BioLegend, 400357) at 1:50 dilution. All staining procedures were performed on ice for 20 minutes.

All flow cytometry experiments were performed using Guava easyCyte flow cytometer (Millipore), and the resulting data were analyzed using FlowJo (FlowJo, RRID:SCR_008520) software. Gates for positive events were set appropriately using untreated and isotype controls as references.

### *In Vitro* Cytotoxicity and Conjugation assay

*In vitro* cytotoxicity assays were performed using flow cytometry, based on a previously reported method with modifications (28). Briefly, human melanoma cells were adjusted to a concentration of 10^6^ cells/mL and were loaded with CellTrace Yellow (Invitrogen, C34567) at 1:500 dilution for 20 minutes at 37°C. Then, cells were washed with complete culture media to remove excess dyes before being seeded at 5 × 10^4^ cells/well in 24-well plates one day ahead of the cytotoxicity assay.

To study the effect of pH on *in vitro* cytotoxicity of NK-92 cells, cells were preincubated in pH-controlled culture media for 24 hours. To study the effect of pharmacologic mTORC1 or ERK inhibition, cells were pretreated with Torin 1 for 4 hours or trametinib for 1 hour, respectively. After incubation, NK-92 cells were counted and added at 3:1 ratio to WM3629 cells seeded as described previously. After 6-hour incubation, cells were dissociated from culture plates using Accumax cell dissociation solution (Sigma, A7089) and analyzed by flow cytometry. The total number of surviving melanoma cells were calculated using the volume of cell mixture, concentration of events, and the percentage of CellTrace Yellow-positive events in all events. Percent survival was calculated by dividing the number of surviving melanoma cells in NK-92–containing wells by the number of surviving melanoma cells in control wells (without NK-92 cells), and percent killing was calculated by 100% − percent survival.

Conjugation assay was performed using flow cytometry as described previously (29). K562 cells as targets were loaded with CellTrace Yellow dye following a similar labeling procedure as described for the cytotoxicity assay. NK-92 cells, which express EGFP from the transduced lentiviral vectors, were mixed with labeled K562 cells at 1:2 ratio for 0 to 60 minutes. At the end of each timepoint, cells were fixed with 2% PFA for 20 minutes at 37°C followed by flow analysis. Percent conjugation was calculated by dividing the number CellTrace Yellow-EGFP double-positive events by the number of all EGFP-positive events.

### Immunoblot Analysis

NK-92 cells were incubated in pH-controlled culture media for 6 or 24 hours. Media were removed by centrifugation, and cells were quickly washed with PBS adjusted to the same pH as culture media to minimize the effect of washing on pH_i_. Total protein from cells was harvested by passive lysis with M-PER Mammalian Protein Extraction Reagent (Thermo Fisher Scientific) supplemented with Protease Inhibitor Cocktail (Promega, G6521), Phosphatase Inhibitor Cocktail 2 and 3 (Sigma, P5726 and P0044). Protein lysates were cleared by centrifugation, quantified using DC Protein Assay (Bio-Rad, 5000111), and resolved by SDS-PAGE gels. Protein from gels was transferred onto nitrocellulose membranes using the iBlot 2 Dry Blotting System (Invitrogen). Membranes were blocked using Intercept (TBS) Blocking Buffer (LI-COR, 927-60001) before incubation with the following primary antibodies: rabbit anti-RHEB (Cell Signaling Technology, 13879S, RRID:AB_2721022, 1:1,000), rabbit anti- pS6K (Thr389; Cell Signaling Technology, 9205S, 1:1,000), rabbit anti-S6K (Cell Signaling Technology, 9204S, 1:1,000), rabbit anti-pS6 (Ser235/236; Cell Signaling Technology, 4858S, RRID:AB_916156, 1:2,000), mouse anti-S6 (Cell Signaling Technology, 2317S, RRID:AB_2238583, 1:1,000), mouse anti-α-tubulin (Sigma, CP06, 1:8,000), mouse anti-NHE1 (Santa Cruz Biotechnology, sc-136239, 1:500), rabbit anti-pERK1/2 (Thr202/Tyr204; Cell Signaling Technology, 4370S, 1:2,000), rabbit anti-ERK1/2 (Cell Signaling Technology, 4695S, 1:1,000), rabbit anti-CA9 (Novus Biologicals, NB100-417, 1:2,000), mouse anti-perforin 1 (Santa Cruz Biotechnology, sc-374346, 1:300), rabbit anti-granzyme B (Cell Signaling Technology, 4275S, 1:2,000), and rabbit anti-c-Myc (Abcam, ab32072, 1:1,000). Secondary antibodies included Alexa Fluor 680-conjugated goat anti-rabbit IgG (H+L) (Invitrogen, A-21109, 1:8,000) and DyLight 800-conjugated goat anti-mouse IgG (Cell Signaling Technology, 5257S, RRID:AB10693543, 1:15,000). All antibodies were diluted in 5% BSA in TBS supplemented with 0.1% Tween 20. Immunoblots were imaged with Odyssey CLx Imaging System (LI-COR). Images were processed with Image Studio software (Image Studio Lite, RRID:SCR_013715) with linear contrast enhancement.

The same membrane might be used to detect multiple proteins by stripping and reblotting. To strip primary and secondary antibodies, membranes were incubated in 1 × NewBlot Nitro Stripping Buffer (LI-COR, 928-40030) for 5 minutes with agitation, followed by blocking as described above.

### Degranulation Assay

Degranulation assay was performed on the basis of a previously reported protocol (30) with modifications. Briefly, 2.5 × 10^4^ NK-92 cells and 5 × 10^4^ target cells (K562 or human melanoma cell lines) in 100 μL of culture media were seeded into 96-well U-bottom plates. If the effect of pH was to be examined, cells were preincubated in pH-controlled media for 24 hours prior to seeding. PE-Cy7–conjugated mouse anti-human CD107a antibody (BD Biosciences, 561348) was added at 5 μL/well to label cell-surface CD107a (LAMP1). After 1-hour incubation, GolgiStop (containing monensin; BD Biosciences, 554724) and GolgiPlug (containing brefeldin A; BD Biosciences, 555029) were added at 4 μL/6 mL and 1 μL/1 mL, respectively, to inhibit the internalization of CD107a. Cells were analyzed using a flow cytometer for PE-Cy7 fluorescence after a total of 6 hours of incubation.

### pH_i_ and NHE1 Activity

pH_i_ of NK-92 cells was measured by staining with SNARF-1, a ratiometric pH indicator dye, based on a similar method reported previously (31). Briefly, NK-92 cells were loaded with 5 μmol/L of 5-(and-6)-carboxy SNARF-1, acetoxymethyl ester, acetate (Invitrogen, C1272) for 20 minutes at 37°C. Excessive dyes were removed by washing the cells with culture media, and cells were allowed to recover in media for 30 minutes at 37°C. The media were then changed to either pH-controlled live-cell imaging buffers (140 mmol/L NaCl, 2.5 mmol/L KCl, 1.8 mmol/L MgCl_2_, 1 mmol/L CaCl_2_, 5 mmol/L D-glucose, 10 mmol/L HEPES, and 10 mmol/L PIPES) or pH-controlled high-potassium buffers from the Intracellular pH Calibration Buffer Kit (Invitrogen, P35379), and cells were allowed to accommodate for 30 minutes at room temperature and atmospheric CO_2_. Cells in high-potassium buffers were further treated with 10 μmol/L of nigericin and 10 μmol/L of valinomycin to equilibrate pH_e_ and pH_i_. Finally, cells were analyzed using a flow cytometer with dual fluorescent emissions captured at around 580 and 640 nm wavelengths. The emission ratio at these two wavelengths for cells in the high-potassium buffers was plotted against the pH of the buffers, and a second-order polynomial fit was used to derive a standard curve for interpolation to determine the baseline pH_i_ of the cells.

NHE1 activity was measured as the rate of pH_i_ recovery after an acute acid load caused by ammonium chloride (NH_4_Cl), as described previously (32). NK-92 cells were first loaded with SNARF-1 following the same procedure as in the measurement of pH_i_. Then, a portion of cells was used to generate a standard curve using high-potassium buffers. The remaining cells were first incubated in isotonic NH_4_Cl solution (50 mmol/L NH_4_Cl, 70 mmol/L choline chloride, 5 mmol/L KCl, 1 mmol/L MgCl_2_, 2 mmol/L CaCl_2_, 5 mmol/L glucose, and 20 mmol/L HEPES-Tris, pH 7.4) for 5 minutes at room temperature and atmospheric CO_2_. Next, an acute acid load was induced by incubating the cells with isotonic Na^+^-free solution (125 mmol/L choline chloride, 1 mmol/L MgCl_2_, 2 mmol/L CaCl_2_, 5 mmol/L glucose, and 20 mmol/L HEPES-Tris, pH 7.4) for 10 minutes. Following the incubation, the solution was replaced by a Na^+^-rich solution (140 mmol/L NaCl, 5 mmol/L KCl, 1 mmol/L CaCl_2_, 1 mmol/L MgCl_2_, 10 mmol/L glucose, and 10 mmol/L HEPES, pH 7.4), and cells were immediately analyzed on a flow cytometer for dual fluorescent emission as in the measurement of pH_i_. pH_i_ recovery was measured for 10 minutes, and the rate of pH recovery was calculated using linear regression based on the data between 2 and 5 minutes.

### Visualization of Immunologic Synapses

To characterize immunologic synapses formed by NK-92 cells, we coated glass coverslips with 5 μg/mL of purified anti-human CD18 (BioLegend, 302102, RRID:AB_314220) and purified anti-human CD337 (NKp30) antibodies for 30 minutes at 37°C. Excess antibodies were washed with PBS, and the coverslips were transferred to a 6-well plate. NK-92 cells were added onto the coverslips at a concentration of 2 × 10^5^ cells/mL and were allowed to adhere and form immunologic synapses for 30 minutes at 37°C. Coverslips with cells were washed with PBS before being treated with 2% PFA for fixation. Fixed cells were permeabilized with 0.1% Triton X-100, blocked with 5% BSA, and stained with 4′,6-diamidino-2-phenylindole (DAPI), Alexa Fluor 568–conjugated phalloidin (Invitrogen, A12380, 1:100), and Alexa Fluor 647–conjugated anti-human perforin antibody (BioLegend, 308110, 1:100) for 1 hour. Coverslips were mounted onto glass slides, and stained cells were visualized under an Axio Observer Z1 inverted microscope (ZEISS) outfitted with a CSU-W1 spinning disc (Yokogawa) with a 100 × objective lens and optical sectioning at 0.25 μm increments from the cell-surface contact site.

We assessed the convergence of perforin-containing lytic granules around the immunologic synapses based on previously reported methods (33). Briefly, we measured the average distance between individual lytic granules and the geometric center (centroid) of all the granules in each cell using a maximum intensity Z-projection image obtained by confocal microscopy. The distance serves as a measurement of the spread of lytic granules in the X-Y direction, as a higher average distance indicates more spread (and less convergence). To assess the spread of lytic granules along the Z-axis, we measured the size of the perforin-positive area on each slice of the Z-stack using the automatic thresholding method Maximum Entropy in the ImageJ software (ImageJ, RRID:SCR_003070 and plotted the normalized area against distance based on slice number. Then, assuming Gaussian distribution of lytic granules along the Z-axis, we performed a Gaussian fit to derive the standard deviation of the perforin-positive area. Alternatively, we calculated the percentage of lytic granules within the first 4 μm above the cell-surface contact plane (first 16 slices) as a separate measurement of lytic granule convergence at the vertical direction relative to the cell-surface contact plane.

### QuantSeq 3′ mRNA Sequencing

Library preparation and sequencing experiments were performed by staff at the Genomics Facility at the Wistar Institute. Libraries for 3′ mRNA sequencing (mRNA-seq) were generated from 100 ng of DNase I-treated total RNA using QuantSeq 3′ mRNA-seq Library Prep Kit FWD (Lexogen), according to the manufacturer's directions. Overall library size was determined using the Agilent TapeStation and the D5000 ScreenTape System (Agilent). Next, libraries were quantified using real-time PCR with kits from Kapa Biosystems. Finally, libraries were pooled for high-output, single-read, 75-bp next-generation sequencing on a NextSeq 500 sequencer (Illumina).

The quality of the sequencing reads was first checked using FastQC. Then, read mapping was performed using Kallisto. Downstream analyses were all performed using R (version 4.1.2) with indicated packages. We first converted the expression levels of genes in raw counts to normalized, log_2_-transformed counts per million mapped reads using edgeR. Differentially expressed genes were identified using Limma-Voom and visualized by heatmap using gplots. Next, we used clusterProfiler to determine the enrichment of transcription binding sites curated in category C3 of the MSigDB database. Finally, we used GSEABase to perform gene set enrichment analysis (GSEA) and plotted the results using enrichplot.

### Statistical analysis

All data with replicates are expressed as mean ± SEM. Unpaired, two-tailed Student *t* test was performed for comparisons involving two groups. One-way or two-way ANOVA followed by Dunnett multiple comparisons test was performed for comparisons involving more than two groups. Linear regression was performed to determine the pH_i_ recovery rate. A second-order polynomial fit was performed to derive the calibration curve in the measurement of pH_i_. A spline fit was performed to obtain an approximation of the pH_e_-pH_i_ curve. A Gaussian fit was performed to assess the spread of perforin-containing granules, with F test to compare the variances across the groups. A *P* value of less than 0.05 was considered statistically significant. Pairwise comparisons are not statistically significant unless marked with asterisks. All statistical analyses were performed using GraphPad Prism 9 (GraphPad Prism, RRID:SCR_002798).

### Data Availability

The raw data for QuantSeq 3′ mRNA-seq were generated at the Genomics Facility at the Wistar Institute and are publicly available in Gene Expression Omnibus at GSE205146. Derived data supporting the findings of this study are available from the corresponding author upon request. All other data generated in this study are available upon request from the corresponding author.

## Results

### Systemic Buffering Negated the Effect of Anti-PD1 Antibody in a Mouse HCC Model

Multiple studies have shown that acidic culture media inhibit the antitumor activity of immune effector cells such as cytotoxic T and NK cells (13, 14). Therefore, we hypothesized that systemic buffering with oral NaHCO_3_ would enhance T- or NK-cell function and may thus synergize with anti-PD1 in treating transgenic mice with inducible *MYC*–driven HCC (27).

**FIGURE 1:**
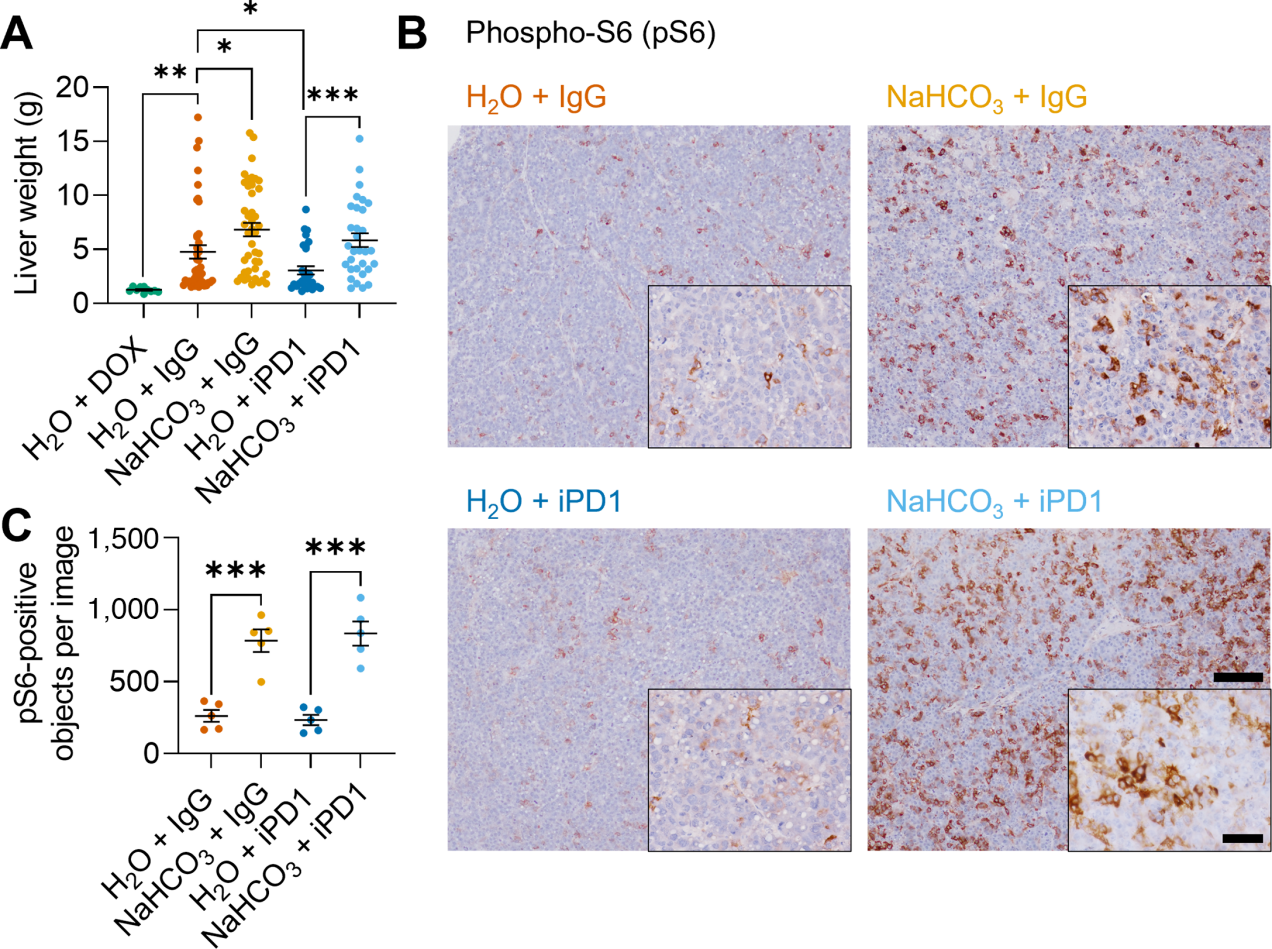
Systemic buffering negated the effect of anti-PD1 antibody in a mouse hepatocellular carcinoma (HCC) model. **A,** Liver weight of mice with doxycycline (DOX)-repressible *MYC*–driven HCC fed with regular or sodium bicarbonate (NaHCO_3_)-containing drinking water and treated with anti-PD1 antibody (iPD1) or isotype control (IgG). Each dot represents an individual mouse liver weight in the different groups, all determined upon euthanasia at 12 weeks after birth (*N* = 10, 43, 43, 30, and 31 animals for H_2_O + DOX, H_2_O + IgG, NaHCO_3_ + IgG, H_2_O + iPD1, and NaHCO_3_ + iPD1 groups, respectively; unpaired *t* test). **B,** Representative microscopic images of the mTORC1 target pS6-stained tumor slices. Scale: 100 μm (inset: 40 μm). **C,** Quantification of pS6-positive objects in images in B (*N* = 5 whole-tumor scans from different animals, one-way ANOVA with multiple comparisons). *, *P* < 0.05; ***, *P* < 0.001.

In this Tet-Off inducible *MYC* model, silencing of *MYC* expression is achieved by the treatment with doxycycline (DOX; Fig. 1A), which prevents HCC tumor formation. After removing DOX to initiate HCC tumorigenesis, we compared various treatments and their effects on tumor formation using liver weight as a surrogate for tumor burden. Unexpectedly, contrary to others’ observation that systemic buffering by NaHCO_3_ synergized with anti-PD1 in their tumor model (15), we found systemic buffering by NaHCO_3_ negated the antitumor effect of anti-PD1 antibody (Fig. 1A) in our model. Furthermore, the accelerated tumor growth in NaHCO_3_-treated mice was concomitant with a remarkable increase in tumor cell mTORC1 activity (Fig. 1B and C), which we observed previously in mice with xenografts of the HCT116 human colon or MCF7 breast cancer cell lines (19). mTORC1 is thought to be a driver of murine HCC, as sustained activation of mTORC1 is sufficient to cause murine HCC (34).

In this instance, systemic buffering had adverse effects, indicating that treatment response depends on specific tumor models and the dependency of tumor cells on mTORC1 activity for tumor growth. Therefore, we focused on metabolically engineering immune effector cells for ACT as an alternative approach to target the adverse effect of acidity within the TME.

### NK-92 Cells as Model Immune Effector Cells for Metabolic Engineering

To study the metabolic engineering of immune effector cells, we sought an experimentally tractable system amenable to genetic manipulations. Unfortunately, primary immune effector cells such as CD8^+^ T cells and NK cells are not readily tractable to manipulate experimentally, expand, and select after genetic manipulation for our proof-of-concept study of metabolic engineering (35). Therefore, we used the IL2-dependent human NK cell line NK-92, which was isolated from a patient with non–Hodgkin lymphoma (20). NK-92 cells resemble activated NK cells but lack most inhibitory receptors, rendering them highly cytotoxic against tumor cells (36). As an attractive off-the-shelf product for allogenic ACT, irradiated NK-92 cells have been tested in several phase I clinical trials for patients with solid tumors, including renal cell carcinoma and lung cancer (37). However, the limited clinical response to date underscores the need for developing modified NK-92 cells with enhanced antitumor activity.

**FIGURE 2:**
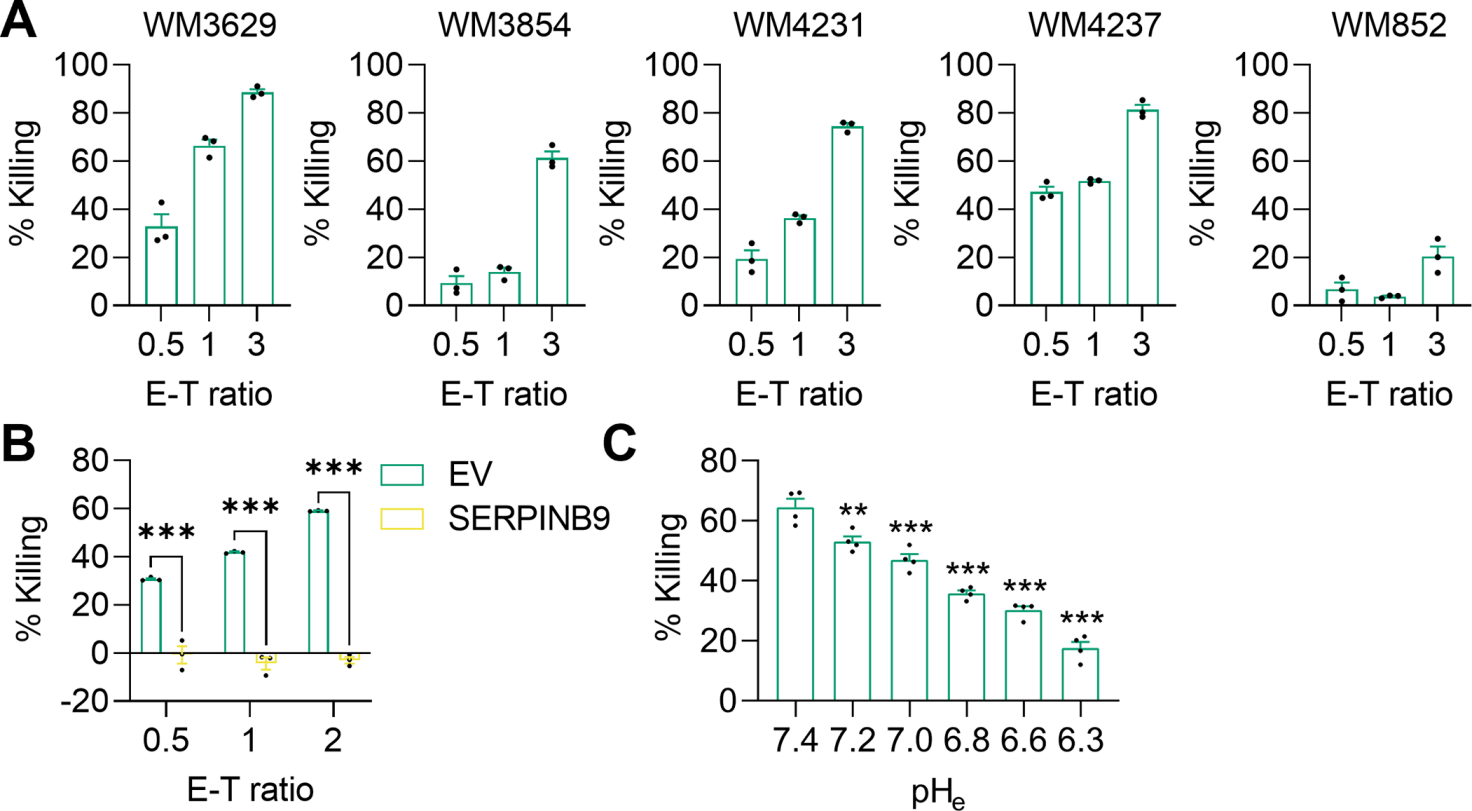
NK-92 cells show cytotoxicity against several human melanoma cell lines *in vitro*. **A,***In vitro* cytotoxicity of NK-92 cells against human melanoma cell lines WM3629, WM3854, WM4231, WM4237, and WM852 at various effector-to-target (E-T) ratios (*N* = 3 wells for each condition). **B,***In vitro* cytotoxicity of NK-92 cells against WM3629 cells overexpressing SERPINB9, a granzyme B inhibitor, at various E-T ratios (E-T ratio = 3:1, *N* = 3 wells, unpaired *t* test). **C,***In vitro* cytotoxicity of NK-92 cells against WM3629 cells in pH-controlled media (defining extracellular pH, or pH_e_; E-T ratio = 3:1, *N* = 4 wells, one-way ANOVA with multiple comparisons versus pH_e_ = 7.4). **, *P* < 0.01; ***, *P* < 0.001.

To develop a tumor model for engineered NK-92 cells, we first tested the *in vitro* cytotoxicity of unmodified NK-92 cells against a panel of previously characterized human melanoma cell lines (22). We observed varying degrees of cytotoxicity depending on the target melanoma cell line (Fig. 2A). Among the human melanoma cell lines tested, we focused on WM3629, an NK-92–sensitive cell line that forms aggressive tumor xenografts in immunodeficient mice (38).

Like cytotoxic T cells, the effector function of NK-92 cells depends on immune synapse formation and the subsequent release of cytolytic proteins such as granzyme B (37). As such, we validated our cytotoxicity assay and documented that overexpression of SERPINB9, a natural inhibitor of granzyme B, in melanoma target cells rendered them resistant to NK-92 cytotoxicity (Fig. 2B). Moreover, as reported for primary NK cells, NK-92 cells also exhibited diminished cytotoxicity in acidic culture media (Fig. 2C). These features of NK-92 cells make them an attractive and tractable ACT model for our study.

### Activating mTORC1 by RHEB Enhances the Cytolytic Activity of NK-92 Cells

As a proof of concept for metabolic engineering, we first sought to restore signaling pathways in immune effector cells affected by acidosis, such as mTORC1, as we reported previously (19). The acidic environment promotes the anterograde trafficking of lysosomes in epithelial cells, which separates lysosomal mTORC1 and perinuclear RHEB, a protein necessary for mTORC1 activation.

**FIGURE 3:**
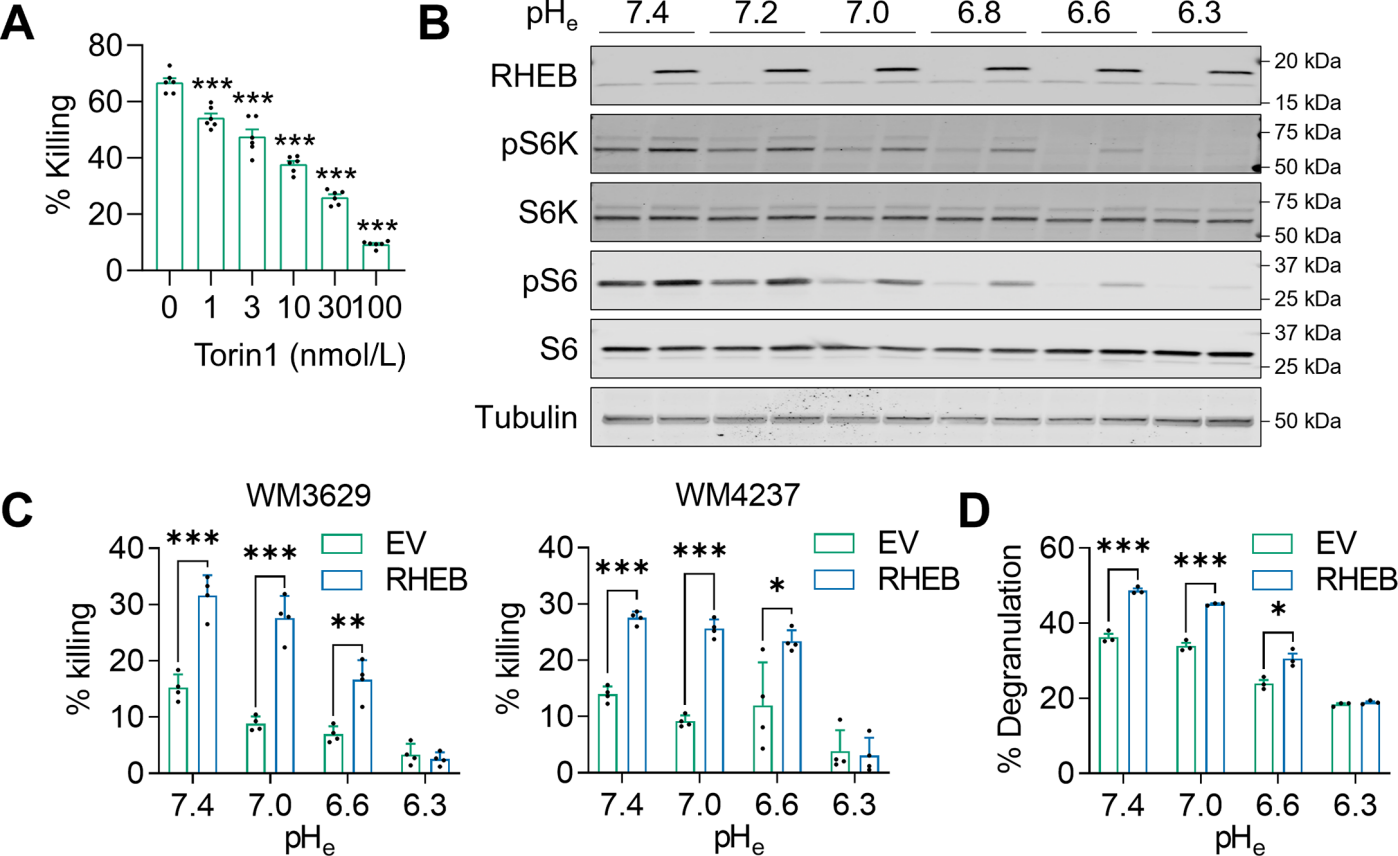
Activating mTORC1 by RHEB enhances cytolytic activity of NK-92 cells. **A,***In vitro* cytotoxicity of Torin1 (mTOR inhibitor)-treated NK-92 cells against the human melanoma cell line WM3629, relative to the untreated cells (E-T ratio = 3:1, *N* = 6 wells, one-way ANOVA with multiple comparisons versus untreated). **B,** Representative immunoblots of the mTORC1 phosphorylation substrates pS6K and pS6 in constitutively active RHEB (RHEB^N153T^, hereafter referred to as RHEB)-overexpressing or EV control NK-92 cells treated with pH-controlled media for 6 or 24 hours. Endogenous RHEB is shown as the dim, lower band in the images, where the bright, upper band represents the overexpressed RHEB. **C,***In vitro* cytotoxicity of RHEB-expressing or EV control NK-92 cells against human melanoma cell lines WM3629 and WM4237 in pH-controlled media (E-T ratio = 3:1, *N* = 4 wells for each cell line, unpaired *t* test). **D,** Degranulation of RHEB-overexpressing or EV control NK-92 cells towards the human leukemia cell line K562 in pH-controlled media (E-T ratio = 1:2, *N* = 3 wells, unpaired *t* test). *, *P* < 0.05; **, *P* < 0.01; ***, *P* < 0.001.

mTORC1 activity is crucial for the effector function of NK cells, as mTORC1 metabolically primes NK cells for effector function (39), and deletion of the mTORC1 component Raptor impairs the effector function of mouse NK cells (40). In addition, we also observed that pretreating NK-92 cells with Torin 1, a selective mTOR inhibitor, reduced their *in vitro* cytotoxicity (Fig. 3A). Therefore, we tested whether we could restore the cytotoxicity of NK-92 cells in an acidic environment by boosting mTORC1 activity. We overexpressed a constitutively active RHEB (RHEB^N153T^, hereafter referred to as RHEB; ref. 24) in NK-92 cells and observed elevated mTORC1 signaling, as indicated by increased phosphorylation levels of mTORC1 targets pS6K and pS6, in the resulting cells at neutral or increasingly acidic pHs (Fig. 3B). RHEB-overexpressing cells also demonstrated enhanced *in vitro* cytotoxicity (Fig. 3C) and degranulation (Fig. 3D) toward tumor cells over the pH_e_ range of 6.6 to 7.4. These observations are consistent with the previous report that overexpressing a wild-type RHEB in primary human CD8^+^ T cells enhanced their cytolytic activity (41). However, as documented, increased mTORC1 activity in CD8^+^ T cells rendered them incapable of transitioning into a memory state, limiting their long-term efficacy (42). Hence, we sought an orthogonal approach to metabolically engineer immune effector cells with proton extruders using NK-92 cells in a proof-of-concept study.

### CA9 does not Rescue the Acid-Blunted Cytolytic Activity of NK-92 Cells

Because restoring the acid-blunted mTORC1 could enhance the cytotoxicity of NK-92 cells, we next determined whether direct metabolic engineering of NK-92 cells by overexpressing acid extruder proteins could rescue their acid-blunted mTORC1 activity and thereby enhance *in vitro* cytotoxicity. Therefore, we first overexpressed CA9 in NK-92 cells. CA9 is a hypoxia-inducible, membrane-bound extracellular CA catalyzing the reversible hydration of CO_2_ at the cell surface and is thought to promote tumor growth by protecting tumor cells from acidosis (10).

To verify that the lentiviral construct expressing CA9 was functional, we first transduced WM3629 human melanoma cells with the CA9-encoding lentivirus. We found that WM3629 cells with overexpressed CA9 had increased pH_i_ (Supplementary Fig. S1A), suggesting that the overexpressed CA9 was functional in these cells. In addition, we found that overexpressing CA9 partially rescued acid-blunted mTORC1 activity in WM3629 cells (Supplementary Fig. S1B). Therefore, we proceeded to overexpress CA9 in NK-92 cells. Surprisingly, overexpressed CA9 failed to elevate baseline pH_i_ (Supplementary Fig. S1C) of NK-92 cells, although it partially rescued the acid-blunted mTORC1 activity in multiple independent experiments (Supplementary Fig. S1D and S1E). Importantly, ectopic CA9 could not consistently enhance NK-92 cytotoxicity (Supplementary Fig. S1C) or degranulation (Supplementary Fig. S1D) toward tumor target cells. The failure of CA9 alone to increase pH_i_ could be because CA9 may require additional proteins, including the intracellular CA2 and sodium bicarbonate cotransporter 1 (NBCE1), to efficiently alkalinize the cytoplasm according to previously proposed models (43). Intriguingly, the lack of sufficient cytoplasmic alkalinization or cytotoxicity by CA9 overexpression was accompanied by increased mTORC1 activity, suggesting that the partially rescued mTORC1 under low pH_e_ was insufficient for enhanced NK-92 cytolytic activity. As such, whether the constitutively active RHEB, which enhanced mTORC1 activity, has an additional function (44) in increasing NK-92 cytotoxicity remains to be established.

### NHE1 Enhances the Cytolytic Activity of NK-92 Cells

As an alternative to CA9, which failed to enhance cytotoxicity, we used the Na^+^/H^+^-exchanger protein NHE1, encoded by *SLC9A1*, to engineer NK-92 cells with the ability to extrude H^+^. NHE1 utilizes the sodium gradient across the plasma membrane of cells to export protons in exchange for sodium influx. Like CA9, NHE1 is also known to regulate pH_i_ and pH_e_ in tumor cells and is thought to promote tumor cell migration by acidifying their periphery (45). Therefore, we hypothesized that NHE1 would enhance the cytolytic activity of NK-92 cells by increasing their pH_i_, which is also expected to increase mTORC1 activity.

**FIGURE 4:**
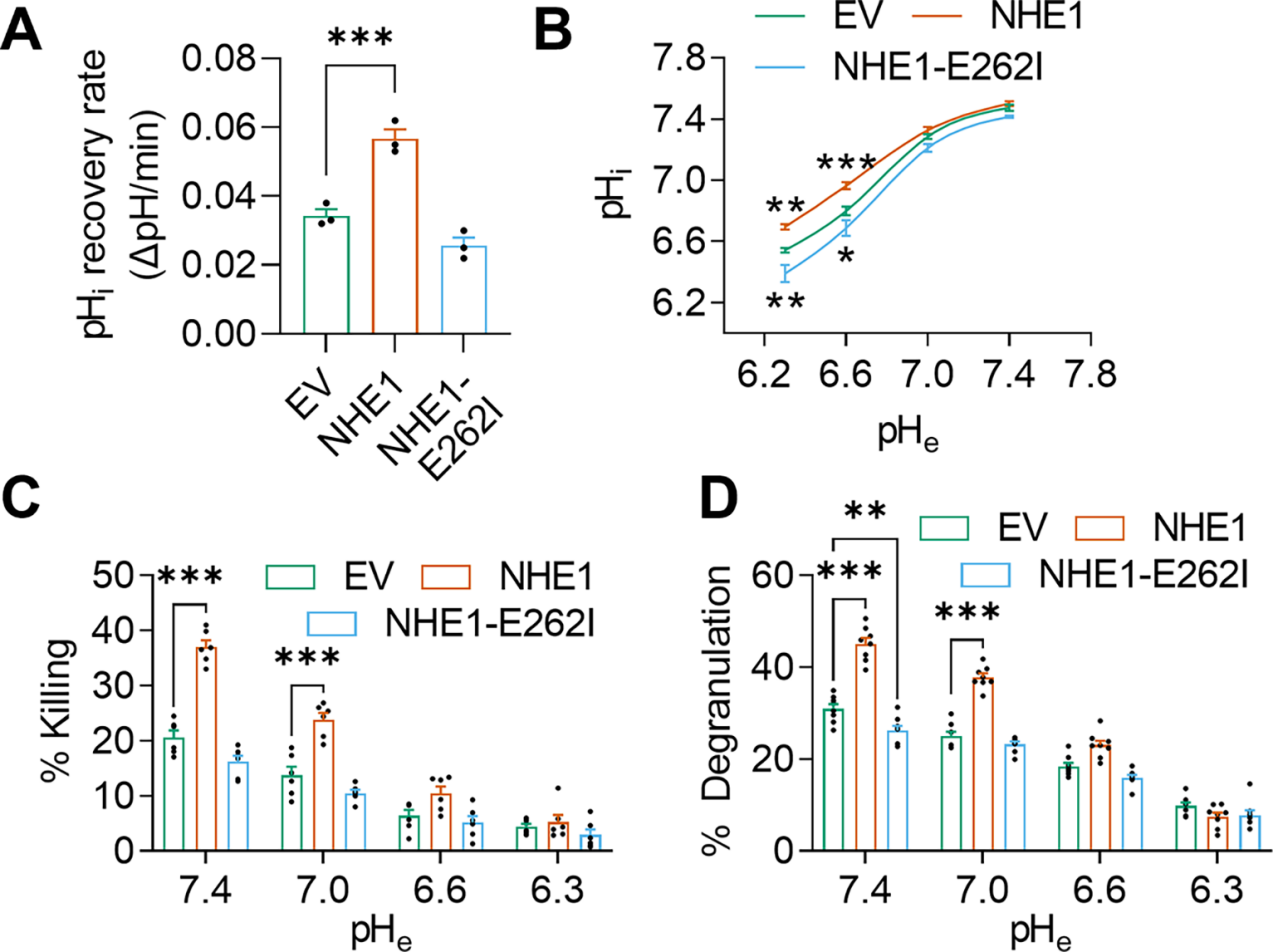
Overexpressing NHE1 enhances the cytolytic activity of NK-92 cells. **A,** pH_i_ recovery rate of NK-92 cells expressing constitutively active NHE1 (hereafter referred to as NHE1), inactive NHE1 (hereafter referred to as NHE1-E262I), or EV after an acute acid load (*N* = 3 samples, spline fit, one-way ANOVA with multiple comparisons). **B,** Baseline pH_i_ of NK-92 cells expressing NHE1, NHE1-E262I, or EV in pH-controlled media (*N* = 6 wells, pooled from two independent experiments, two-way ANOVA with multiple comparisons, smooth connecting curve generated by second-order polynomial fit). **C,***In vitro* cytotoxicity of NK-92 cells expressing NHE1, NHE1-E262I, or EV against the human melanoma cell line WM3629 in pH-controlled media (E-T ratio = 3:1, *N* = 6 wells, two-way ANOVA with multiple comparisons). **D,** Degranulation of NK-92 cells expressing NHE1, NHE1-E262I, or EV towards K562 cells in pH-controlled media (E-T ratio = 1:2, *N* = 8 wells, pooled from two independent experiments, two-way ANOVA with multiple comparisons). *, *P* < 0.05; **, *P* < 0.01; ***, *P* < 0.001.

We synthesized the codon-optimized cDNA of human NHE1 with histidine-to-arginine mutations at the pH-sensitive histidine clusters (Supplementary Table S1), making the resulting NHE1 constitutively active (hereafter referred to as NHE1) regardless of environmental pH (25). As a control, we generated a mutant NHE1 with E262I mutation that ablates ion exchanger activity (hereafter referred to as NHE1-E262I; ref. 46). We found that NK-92 cells overexpressing the constitutively active NHE1 showed faster recovery of pH_i_ from an acute acid load, a standard measurement of NHE1 activity, compared with empty vector (EV)- or inactive NHE1-expressing cells (Fig. 4A). This faster pH_i_ recovery illustrates that the ectopically expressed, constitutively active NHE1 has ion exchanger activity in NK-92 cells. In addition, NK-92 cells expressing NHE1 had a higher baseline pH_i_ than cells overexpressing the inactive mutant NHE1 or EV (Fig. 4B). These cells also showed heightened *in vitro* cytotoxicity (Fig. 4C; Supplementary Fig. S2A) and degranulation as measured by CD107a surface expression (Fig. 4D; Supplementary Fig. S2B). Notably, the higher cytotoxicity of NHE1-expressing NK-92 was not due to altered proliferation (Supplementary Fig. S2C) or survival (Supplementary Fig. S2D). In the 6-hour time frame of the cytotoxicity and degranulation assays, survival is an important factor for consideration. The proliferation rate *in vitro* was measured in consideration of the potential ability of NK-92 to function *in vivo*, which required a much longer time than the *in vitro* assays. The inability of the inactive NHE1 to increase cytotoxicity or degranulation suggests that the ion exchanger activity of NHE1 is required for enhancing the cytolytic activity of NK-92 cells, ruling out other putative noncatalytic functions of NHE1.

### The Increased Cytolytic Activity of NHE1-expressing NK-92 Cells does not Correlate with the Activation of mTORC1 or ERK Pathways

**FIGURE 5:**
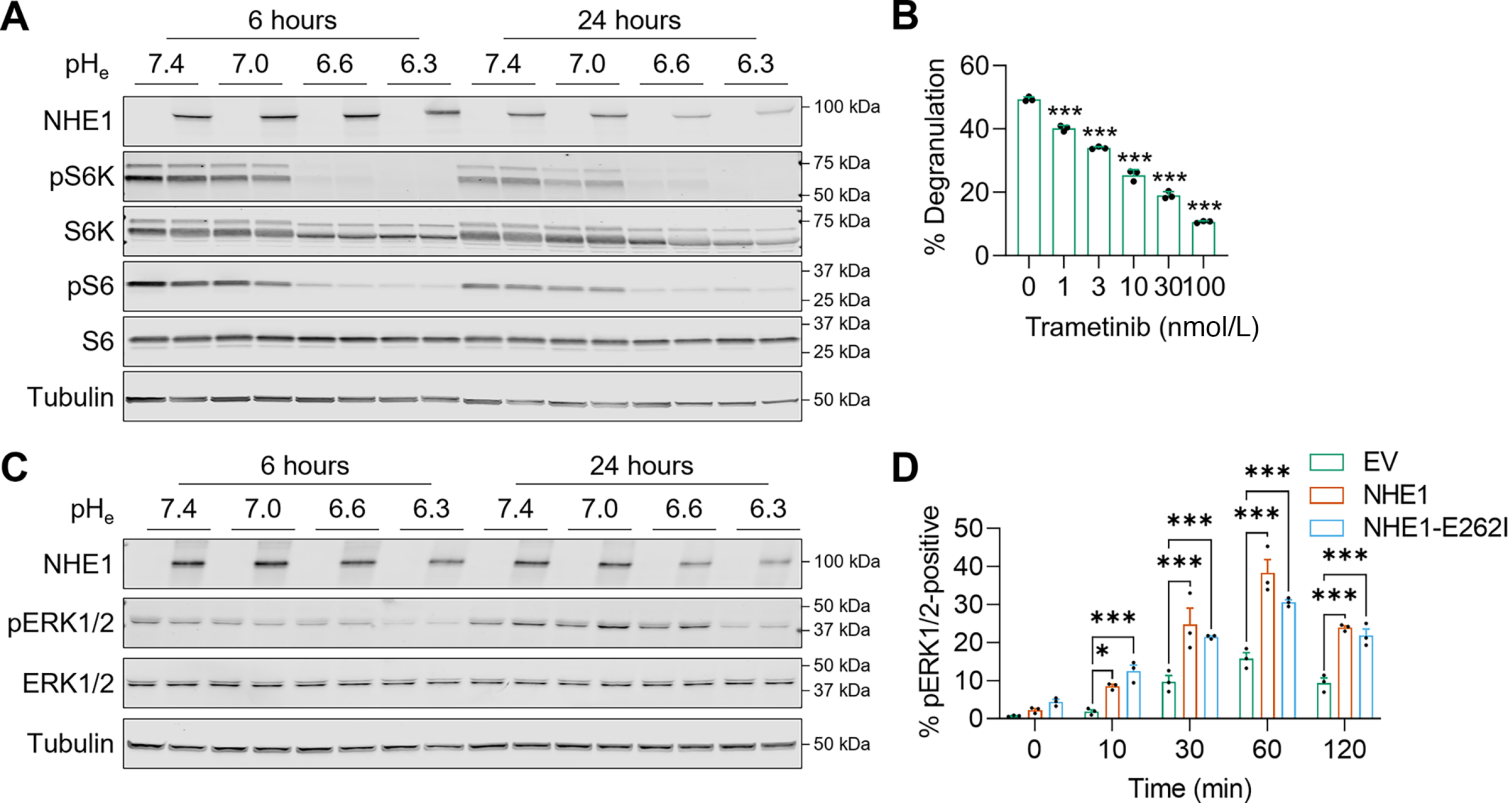
The increased cytolytic activity of NHE1-expressing NK-92 cells does not correlate with the activation of mTORC1 or ERK pathways. **A,** Representative immunoblots assessing mTORC1 substrates pS6K and pS6 in NK-92 cells expressing constitutively active NHE1, NHE1-E262I, or EV treated with pH-controlled media for 6 or 24 hours. At the exposure shown, endogenous NHE1 is barely visible due to low expression. **B,** Degranulation of trametinib (MEK inhibitor)-treated NK-92 cells against K562 cells, relative to untreated cells (E-T ratio = 1:2, *N* = 3 wells, one-way ANOVA with multiple comparisons vs. untreated). **C,** Representative immunoblots assessing phospho-p44/42 MAPK (pERK1/2) in NK-92 cells expressing NHE1, NHE1-E262I, or EV treated with pH-controlled media for 6 or 24 hours. At the exposure shown, endogenous NHE1 is barely visible due to low expression. **D,** Percentage of NHE1-, NHE1-E262I-, or EV-expressing NK-92 cells positive for intracellular pERK1/2 overtime after interacting with K562 cells (E-T ratio = 1:1, *N* = 3 wells, two-way ANOVA with multiple comparisons). *, *P* < 0.05; ***, *P* < 0.001.

We next investigated the mechanisms by which NHE1 enhances the cytolytic activity of NK-92 cells. We started by assessing the activity of acid-blunted signaling pathways that can influence NK-cell effector functions, such as the mTORC1 pathway. We have shown that the acidic environment blunts mTORC1 activity (19) and that activating mTORC1 by overexpressing RHEB (Fig. 3B) enhances the cytotoxicity and degranulation of NK-92 cells (Fig. 3C and D). Therefore, we hypothesized that NHE1 could rescue acid-blunted mTORC1 activity in NK-92 cells by elevating pH_i_, leading to the enhanced cytolytic activity of the cells. Surprisingly, while NHE1 raised the basal pH_i_ of NK-92 cells, it did not rescue the acid-blunted mTORC1 activity (Fig. 5A) as hypothesized. This result, combined with the previous observation that CA9, while partially rescuing mTORC1 activity, did not enhance the cytolytic activity of NK-92 cells (Supplementary Fig. S1C and S1D), suggests that elevated mTORC1 is not necessary for enhanced degranulation or cytotoxicity.

Because NHE1-overexpressing NK-92 cells demonstrated elevated degranulation, we next assessed the activity of MAPK pathways in the cells, as others have reported that phosphorylation of ERK is required for the proper orientation of lytic granules in NK cells during degranulation (47). In addition, ERK has been shown to physically interact with the ectopically expressed NHE1 (48). Congruent with this notion, inhibiting MEK, an upstream kinase of ERK, in NK-92 cells reduced their degranulation (Fig. 5B). However, while we observed increased basal and target cell–induced ERK phosphorylation in NK-92 cells expressing the constitutively active NHE1 (Fig. 5C and D), the catalytically inactive mutant NHE1 also increased target cell–induced ERK phosphorylation (Fig. 5D) without enhancing cytotoxicity (Fig. 4C). These results suggest that ERK phosphorylation is not sufficient to enhance NK-92 cytotoxicity, but it may be necessary along with other factors to increase degranulation and cytotoxicity.

### NHE1 Promotes Degranulation of NK-92 Cells

**FIGURE 6:**
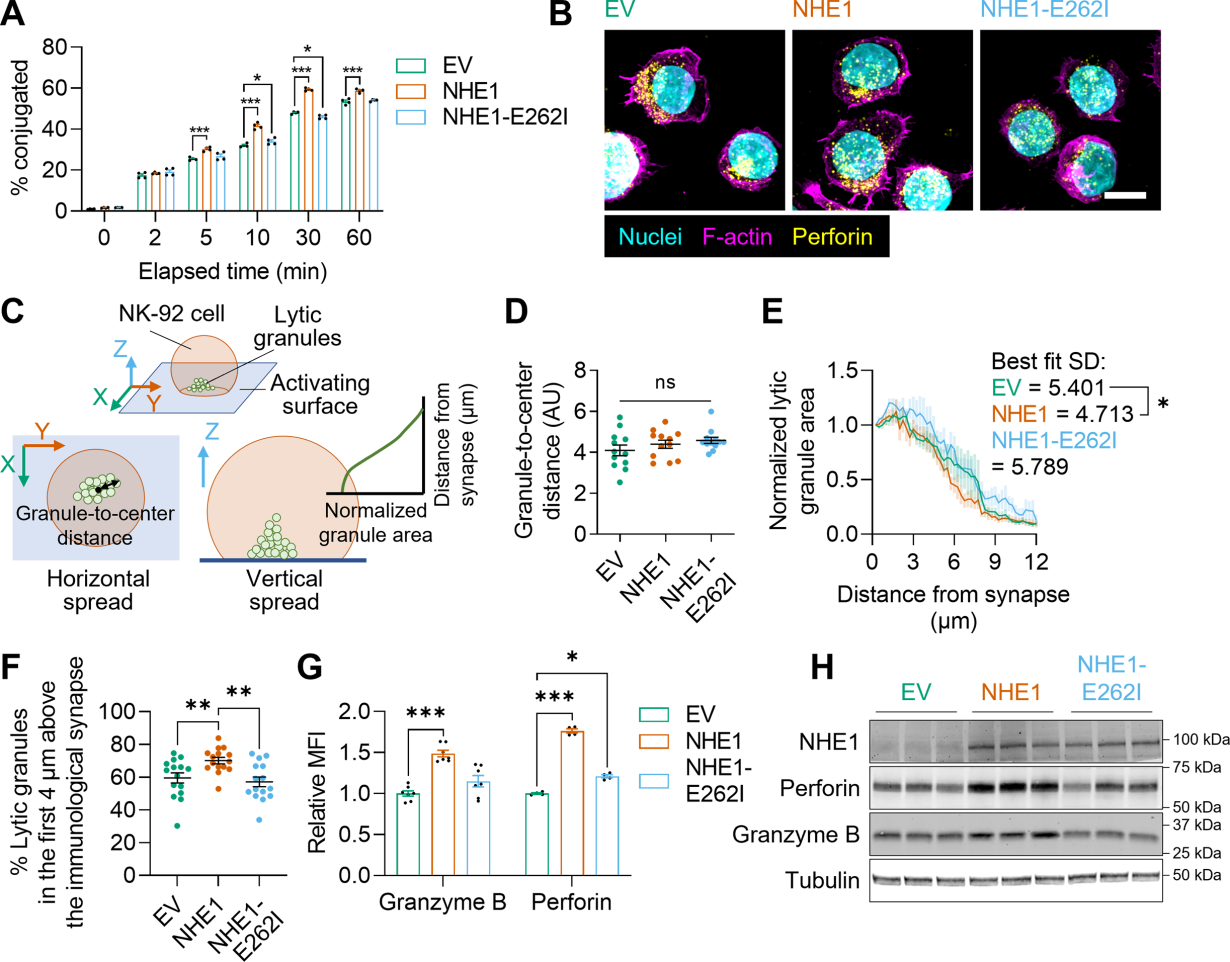
NHE1 promotes degranulation of NK-92 cells. **A,** Conjugate formation between NK-92 cells expressing NHE1, NHE1-E262I, or EV and K562 cells over time (E-T ratio = 1:2, *N* = 4 wells, *X* axis in log_2_ scale, two-way ANOVA). **B,** Representative maximum intensity Z-projection images of antibody-activated NK-92 cells expressing NHE1, NHE1-E262I, or EV, with fluorescent labeling of nuclei (DAPI, cyan) and F-actin (phalloidin, magenta), and immunofluorescence of perforin (yellow). Scale: 10 μm. **C,** Schematic representation of the horizontal (X-Y) and vertical (Z) spread of lytic granules around the immunologic synapse formed between NK-92 cells and antibody-coated activating surface. **D,** Average X-Y distance of perforin-positive lytic granules to their geometric center within NK-92 cells expressing NHE1, NHE1-E262I, or EV in arbitrary units (AU) (*N* = 15 cells from 4 wells). **E,** Distribution of areas of perforin-positive lytic granules across the Z-axis in NK-92 cells expressing NHE1, NHE1-E262I, or EV fitted with a Gaussian curve, with SD from the calculated best fit (F test) (*N* = 15 cells from 4 wells, shaded bars represent SEM). **F,** Percentage of perforin-positive lytic granules within the first 4 μm above the cell-surface contact plane (*N* = 15 cells from 4 wells, one-way ANOVA with multiple comparisons). **G,** Intracellular granzyme B and perforin levels in NHE1- or NHE1-E262I–expressing NK-92 cells relative to EV controls (granzyme B: *N* = 7 wells, pooled from two independent experiments, perforin: *N* = 4 wells, one-way ANOVA with multiple comparisons). **H,** Representative immunoblots of perforin and granzyme B in NK-92 cells expressing EV, NHE1, or NHE1-E262I at basal conditions. *, *P* < 0.05; **, *P* < 0.01; ***, *P* < 0.001.

Degranulation is essential for cytotoxicity. Because NHE1-overexpressing NK-92 cells demonstrated increased degranulation (Fig. 4D), we focused our mechanistic study on the characteristics of degranulation. The superior level of degranulation conferred by NHE1 may reflect the enhanced interaction between NK-92 cells and their target cells. Indeed, NHE1-overexpressing NK-92 cells were more likely to form conjugates with target cells than EV or NHE1-E262I cells (Fig. 6A).

Given the increased degranulation and target cell conjugation conferred by ectopic NHE1, we sought to determine whether there were differences in immunologic synapses formed by NHE1-expressing or EV NK-92 cells. As noted previously, low pH_e_ can lead to low pH_i_, which induces the polarization of lysosomes away from the microtubule-organizing center (MTOC) in epithelial cells (19). Here, we surmise from this observation that by analogy, cytotoxic granules in NK-92 cells, which must move toward the MTOC for degranulation, have diminished ability to move toward the immunologic synapse in acidic conditions.

Therefore, we studied the immunologic synapses formed by NK-92 cells on an activating antibody-coated surface (Fig. 6B; Supplementary Fig. S3). For this assay, NK cell–activating anti-human CD337 (NKp30) antibodies were coated on glass coverslips, and then NK-92 cells were allowed to adhere to the activating surface with the help of anti-human CD18 antibodies. Specifically, we assessed the convergence of lytic granules toward the immunologic synapses formed at the cell-glass contact surface, which promotes the focused release of cytotoxic proteins and efficient killing of target cells by NK cells (33). Using confocal microscopy with optical sectioning, we determined the horizontal and vertical spread of lytic granules around the immunological synapses of NK-92 cells (Fig. 6C). To assess the horizontal spread of lytic granules in the direction parallel to the cell-surface contact plane, we first identified the geometric center (centriole) of perforin-positive lytic granules using the maximum intensity Z-projection images. Then, we calculated the average distance of individual lytic granules to the geometric center as a measurement of the horizontal spread of the lytic granules (Fig. 6C, bottom left). To assess the vertical spread of lytic granules in the direction perpendicular to the cell-surface contact plane, we used two different measurements. First, we measured the area representing perforin-containing lytic granules within each slice of the Z-stack images using autothreshold methods. We plotted the obtained area against the distance of the slices from the cell-surface contact plane (immunologic synapse). Assuming a normal distribution of lytic granules from the contact plane, we fitted the plotted curve with a Gaussian curve and calculated the best fit SD of the resulting Gaussian curve. A larger SD is assumed to represent more distributed lytic granules along the vertical direction (Fig. 6C, bottom right). Alternatively, we calculated the percentage of lytic granules within the first 4 μm above the cell-surface contact plane within each cell. A higher percentage of lytic granules within the immediate layers adjacent to the immunologic synapse indicates higher synaptic convergence in the vertical direction. Although we did not identify any significant differences in the horizontal spread of lytic granules away from their geometric center among NK-92 cells expressing NHE1, NHE1-E262I, or EV (Fig. 6D), we noticed the reduced vertical spread of lytic granules in NK-92 cells expressing the constitutively active NHE1 (Fig. 6E). These cells also have more percentage of lytic granules placed around the immunologic synapse (Fig. 6F). Therefore, we surmise that NHE1 increases the polarization of lytic granules toward the immunologic synapse, thereby enhancing cytotoxicity. These observations are consistent with the previous report that low pH_i_ drives the polarization of lysosomes away from the MTOC, as seen in epithelial cells (19).

Furthermore, we found by flow cytometry and immunoblotting that the levels of cytotoxic proteins perforin and granzyme B were increased in NK-92 cells expressing the constitutively active NHE1 but not the inactive NHE1 (Fig. 6G and H). A potential regulator for the expression of these cytotoxic proteins is c-Myc, as knockout of c-Myc impaired the protein expression of granzyme B in primary mouse NK cells (49). In addition, loss of c-Myc also conferred decreased effector activity of the NK cells (49). Therefore, we hypothesized that NK-92 cells expressing the constitutively active NHE1 had elevated c-Myc activity. Therefore, we performed QuantSeq 3′ mRNA-seq to assess c-Myc–related transcriptomic changes in NHE1-expressing NK-92 cells compared with EV cells. We identified 67 genes that were significantly differentially expressed between NHE1 and EV cells at neutral pHe with a greater-than-2-fold change and *P*_adjusted_ < 0.05 (Supplementary Fig. S4A; Supplementary Table S2). While we did not observe increased transcription of the *MYC* gene (Supplementary Fig. S4B), we found increased protein expression of c-Myc in NHE1-expressing NK-92 cells compared with EV or NHE-E262I cells (Supplementary Fig. S4C). Furthermore, GSEA suggested significant enrichment of potential c-Myc target genes in NHE1-expressing NK-92 cells compared with EV cells (Supplementary Fig. S4B and S4C). In addition, the gene set related to ribosomal biogenesis, a process promoted by c-Myc (50), was also significantly enriched in NHE1-expressing cells (Supplementary Fig. S4B and S4D). Taken together, these results indicate that the increased cytotoxicity of NHE1-overexpressing NK-92 cells is likely due to increased levels of granzyme B and perforin as well as enhanced lytic granule polarization, target cell engagement, and degranulation.

### NHE1 Enhances the Antitumor Activity of NK-92MI Cells *In Vivo*

**FIGURE 7:**
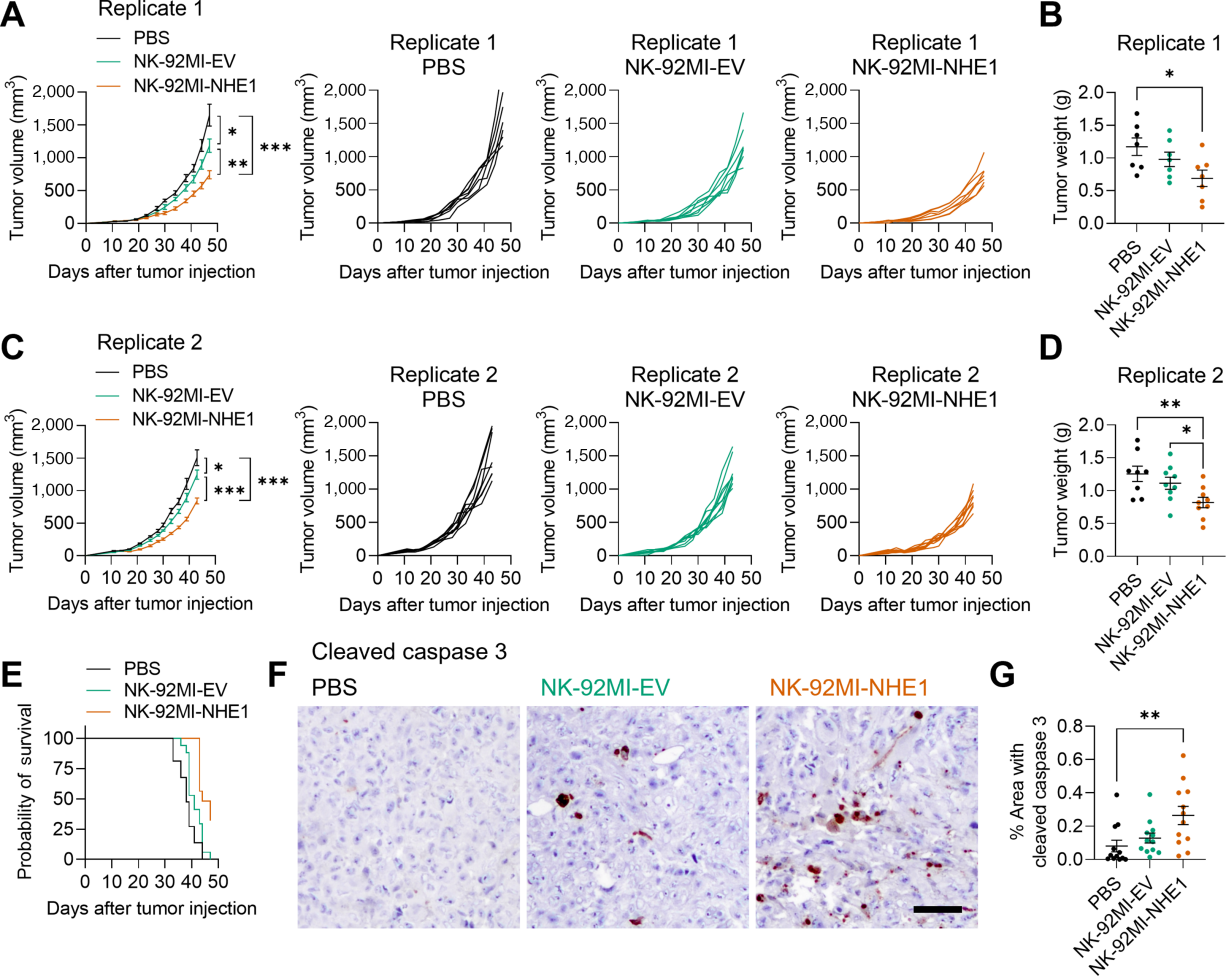
NHE1 enhances *in vivo* cytotoxicity of NK-92MI cells. **A** and **C**, Average and individual tumor volume of mice with WM3629 xenografts treated with intravenous PBS (no cell transfer control) or NK-92MI cells expressing constitutively active NHE1 or EV control (*N* = 7 animals for all groups in **A**, *N* = 8, 9, and 9 animals for PBS, EV, and NHE1 groups in **B**, respectively; two-way ANOVA). **B** and **D**, Tumor weight of the mice in **A** and **C** at endpoints (*N* = 7 for all groups in **B**, *N* = 8, 9, and 9 for PBS, EV, and NHE1 groups in **D**, respectively; unpaired *t* test). **E,** Pooled Kaplan–Meier survival curve of mice treated with PBS, NK-92MI-EV, or NK-92MI-NHE1, where a tumor volume greater than 750 mm^3^ is considered “dead” (*N* = 15, 16, and 16 animals for PBS, EV, and NHE1 groups, respectively; pooled from two independent experiments). **F,** Representative microscopic images of cleaved caspase 3-stained tumor slices from mice treated with PBS or NK-92MI cells expressing NHE1 or EV. Scale: 50 μm. **G,** Quantification of the percentage of cleaved caspase 3-positive area in images in E (*N* = 12 random fields from 3 mice for each group, one-way ANOVA with multiple comparisons). *, *P* < 0.05; **, *P* < 0.01; ***, *P* < 0.001.

Having observed the enhanced cytotoxicity of NHE1-overexpressing NK-92 cells *in vitro*, we proceeded to test the effect of NHE1 on the antitumor activity of NK-92 cells in an animal model. Instead of the conventional NK-92 cells, which are IL2 dependent, we used NK-92MI cells, which stably express human IL2 and were reported to have better performance *in vivo* (21). We confirmed that NHE1-overexpressing NK-92MI cells had increased cytotoxicity *in vitro* compared with the EV control (Supplementary Fig. S5). We treated NSG mice bearing WM3629 xenografts with either NHE1-overexpressing NK-92MI cells, EV control cells, or a no cell transfer control (PBS) by intravenous injection. We observed in two independent experiments that ACT with NK-92MI cells reduced tumor growth compared with no cell transfer controls (Fig. 7A–D). However, NHE1-overexpressing NK-92MI cells were statistically significantly more effective than EV cells at slowing tumor growth and reducing tumor weight at the endpoint of the experiments (Fig. 7B and D). While we did not compare the survival of animals treated with PBS, NK-92MI-EV, or NK-92MI-NHE1 due to animal welfare regulations at the Wistar Institute, we generated a pseudosurvival curve by considering animals with a tumor volume of greater than 750 mm^3^ as “dead” (Fig. 7E), which shows that NK-92MI-NHE1 could limit tumor burden. Finally, we observed increased apoptosis in tumors treated with NHE1-expressing NK-92MI cells (Fig. 7F and G). Consistent with the increased *in vitro* target engagement and cytotoxicity, overexpression of NHE1 can enhance adoptively transferred NK-92 cell function *in vivo* and reduce tumor growth.

## Discussion

Although ACT has transformed cancer immunotherapy with successful applications such as CAR T-cell therapy, many patients with cancer, especially those with solid tumors, remain unresponsive to such therapies (4). A significant challenge to ACT is the immunosuppressive TME, with acidity being a likely metabolic factor limiting the antitumor activity of immune effector cells. Targeting the acidic TME is thus a promising approach to enhance the efficacy of immunotherapy. One method to target the acidic TME is the oral administration of NaHCO_3_, which leads to elevated blood pH and has been shown by MRI to increase intratumoral pH *in vivo* (51). Furthermore, systemic neutralization by bicarbonate boosted the efficacy of ICB or ACT in some animal models (14, 15). Given the clinical observation that NaHCO_3_ enhanced the antitumor activity of transarterial chemoembolization in patients with HCC (52), we sought to determine whether systemic buffering would enhance the effect of the anti-PD1 antibody using an aggressive *MYC*–driven HCC mouse model (18). Surprisingly, we found that NaHCO_3_ increased mTORC1 activity and seemingly negated the effect of the anti-PD1 antibody in curbing tumor growth. In this case, the enhanced tumor mTORC1, sufficient to drive transgenic mouse HCC tumorigenesis (34), is surmised to allow tumor growth to dominate over any potential beneficial effect on antitumor immunity. In addition, the application of systemic buffering could be confounded by the inability to achieve sustained neutralization of tumor pH_e_ due to the rapid restoration of blood pH by the kidney (53), and elevated serum bicarbonate has been shown to decrease respiration, which is harmful to patients with lung disease (54). As such, systemic buffering as an anticancer approach cannot be expected to be generalizable. Hence, we sought to approach tumor acidity by metabolic engineering of immune effector cells. This targeted approach reduces the risk of increasing tumor mTORC1 activity and promoting tumor growth through systemic buffering strategies.

We reasoned that if low pH blunts mTORC1, which is required for T- and NK-cell activity (39, 40), then increasing mTORC1 activity by expressing a constitutively active RHEB would enhance NK-92 cell cytotoxicity. Indeed, we found that the RHEB-mediated increase in mTORC1 could enhance degranulation and *in vitro* cytotoxicity of NK-92 cells. Given this observation, we hypothesized that increasing pH_i_ would increase mTORC1, thereby enhancing the cytotoxicity of NK-92 cells. We hypothesize that the ectopic expression of acid extruder proteins, which are known to alkalinize the cytoplasm of cells, would enhance pH_i_, increase mTORC1 activity, and elevate cytotoxicity of NK-92 cells. We then tested the effects of ectopic expression of acid extruder proteins CA9 and NHE1 in NK-92 cells as an ACT model.

As an acid extruder protein that removes intracellular acid, NHE1 alkalinizes the pH_i_ of NK-92 cells when overexpressed. Others have also suggested that NHE1 was involved in the alkalinization of the cytoplasm of tumor cells (45). Although we have shown that epithelial cells in an acidic environment have lowered pH_i_ and blunted mTORC1 activity (19), NHE1-expressing NK-92 cells, despite having heightened pH_i_, did not show elevated mTORC1 activity. Conversely, we found that ectopic expression of the acid extruder protein CA9 did not increase pH_i_ but elevated mTORC1 without enhancing NK-92 cytotoxicity. Whether and how mTORC1 affects lytic granule function in NK cells is unknown and remains to be established. However, it is possible that mTORC1 activity in NK-92 cells, rather than only responding to pH_i_, responds additionally to changes in pH_e_ with the help of cell surface pH-sensing proteins such as pH-sensing G-protein coupled receptors (GPCRs) (55). Regardless of how acidic pH_e_ is mechanistically sensed to reduce mTORC1 activity, the level of mTORC1 activity in NK-92 cells appears to be dissociated from cytolytic activity, which may depend on other effects of pH_i_ on lytic granule biology.

Although NHE1 does not activate mTORC1 in NK-92 cells, we found that it activated ERK. It has been reported that NHE1 colocalizes with ERK (48) and can be phosphorylated by ERK at its cytoplasmic tail, activating its ion exchanger activity (56). Interestingly, a recent study suggested the existence of a positive feedback loop between the phosphorylation of NHE1 and ERK in BRAF^V600E^-positive glioblastoma cells (57). Consistent with the finding in the previous work that pharmacologic inhibition of NHE1 did not completely block ERK phosphorylation, we showed that a mutant NHE1 without ion exchanger activity still activated ERK. Further studies are needed to elucidate whether such a positive feedback loop exists in immune effector cells and whether the constitutively active NHE1 used in our study maintains a high phosphorylation level.

Mechanistically, our results suggest that NHE1 promotes the degranulation of NK-92 cells toward target cells. Degranulation is a crucial component of the effector function of NK cells and cytotoxic T cells, during which lytic granules containing cytotoxic proteins are released to lyse target cells. While the exact mechanism for NHE1 to affect degranulation is not fully understood, our results suggest two possible explanations. First, NHE1 may alter the trafficking of lytic granules toward target cells, thereby altering the formation of immunologic synapses, as we observed. Supporting this notion is the trend of reduced vertical spread of lytic granules in NK-92 cells expressing NHE1. The trafficking of lytic granules during the degranulation of NK cells involves the concerted reorganization of actin and microtubule cytoskeletons and the activity of their corresponding motor proteins (58). NHE1 is known to physically interact with the ERM family of actin-binding proteins (59) and regulate cellular motility in an ion exchange–dependent manner (60). However, whether NHE1 alters the actin-dependent trafficking during the degranulation of NK cells remains undetermined. We also reported previously that intracellular alkalinization led to microtubule-based translocation of lysosomes toward the MTOC (19), which is in the same direction as lytic granule movements during degranulation. Therefore, it is also possible that NHE1 potentiates NK-92 degranulation by promoting the trafficking of lytic granules toward target cells. However, further studies are needed to understand how the microtubule trafficking system senses pH_i_. For example, albeit technically challenging, genetic manipulation of kinesins or dyneins in NK-92 cells may provide mechanistic insight into how NHE1 may affect lytic granule trafficking as was performed in epithelial cells (19). Second, NHE1 may prepare NK cells for effective degranulation by promoting the accumulation of cytotoxic proteins such as granzyme B and perforin. The exact molecular mechanism leading to the increased expression of these proteins requires further investigation.

As a regulator of pH_i_, NHE1 may also modulate metabolic processes sensitive to pH_i_. For example, it has been predicted that higher pH_i_ promotes oxidative and glycolytic metabolism in metabolically active cells such as cancer cells (61). Interestingly, a recent study showed that pH_i_ alkalinization conferred by acid extruder proteins such as MCT4 or NHE1 was sufficient to promote carbon metabolism in normal hematopoietic cells and leukemia cells (62). In addition, we observed evidence of increased *MYC* activity with the enrichment of *MYC* target genes, including those involved in regulating ribosomal biogenesis (50) in NHE1-expressing cells. Importantly, *MYC* activity is documented to be essential for NK-cell metabolic and functional responses in mice (49). We, therefore, speculate that NHE1-expressing NK-92 cells might have elevated *MYC* activity, which conferred them better functional responses and an advantage in the metabolically challenging TME *in vivo*.

Our study provides mechanistic support for the metabolic engineering of immune effector cells in ACT. Others have already demonstrated the technical feasibility of coexpressing a CAR and a second protein, such as a chemokine, in T cells for enhanced antitumor activity (63). Genetic engineering methods such as the Sleeping Beauty transposon system have also enabled the efficient expression of large transgenes in primary T cells (64). Therefore, it would be possible to coexpress metabolic regulators, such as NHE1, with CAR in T cells or NK cells to help them overcome the immunosuppressive effect of acidity within the TME. Our choice of overexpressing a constitutively active NHE1 offers two advantages for use in CAR T cells. First, the constitutively active NHE1 bypasses the pH sensing of wild-type NHE1, thereby allowing sustained activity of NHE1 while eliminating the need of overexpressing additional proteins involved in pH sensing, such as CHP1 (32). Second, while we and others have shown that directly activating mTORC1 in immune effector cells boosts their cytotoxicity, sustained activation of mTORC1 prevents memory differentiation of therapeutic T cells, potentially limiting their long-term efficacy (42). Our results suggest that NHE1 does not activate mTORC1 in NK-92 cells. However, more research is needed to assess the effect of NHE1 on T-cell differentiation before applying it to CAR T cells. A systematic assessment of animals receiving NHE1-expressing immune effector cells with higher antitumor activity is necessary to determine the safety profile of such cells before applying them to clinical settings.

The current study has several limitations. First, we used NK-92 cells as a model for immune effector cells. While NK-92 cells share similar killing mechanisms as primary NK cells, it remains undetermined whether the effect of overexpressing NHE1 can be reproduced in primary NK cells. Furthermore, despite the documented similarity between NK and cytotoxic T cells regarding effector functions and intracellular signaling (65), it will be necessary to test whether NHE1 enhances the antitumor activity of cytotoxic T cells. Second, while we observed a correlation between increased antitumor activity and enhanced degradation in NHE1-overexpressing NK-92 cells, the exact mechanism underlying such a correlation remains to be conclusively determined. Hence, future studies are necessary to focus on the mechanisms by which NHE1 affects granule trafficking and protein synthesis, which likely contribute to the elevated cytotoxicity of NHE1-expressing NK-92 cells.

In summary, these findings demonstrate the feasibility of metabolic engineering immune effector cells to overcome inhibition in the TME. Furthermore, the metabolic engineering approach can potentially be combined with ACT to enhance its efficacy against solid tumors, where the TME is a significant cause of immune inhibition.

## Supplementary Material

Supplementary Figures 1-5, Tables 1-2 Supplementary Fig. S1. CA9 fails to enhance the cytolytic activity of NK-92 cells in vitro. Supplementary Fig. S2. NHE1 enhances in vitro cytotoxicity of NK-92 cells without affecting proliferation or survival. Supplementary Fig. S3. Representative three-dimensional (3D) reconstruction of confocal microscopic images of antibody-activated NK-92. Supplementary Fig. S4. NHE1 increases the protein expression of c-Myc in NK-92 cells. Supplementary Fig. S5. NHE1 enhances in vitro cytotoxicity of NK-92MI. Supplementary Table S1. Nucleotide sequence of codon-optimized, constitutively active human NHE1 cDNA. Supplementary Table S2. Differentially expressed genes between Na+/H+-exchanger 1 (NHE1)-expressing and empty vector NK-92 cells, ranked by log2(fold change).

## References

[1] Chai LF , Prince E, Pillarisetty VG, Katz SC. Challenges in assessing solid tumor responses to immunotherapy. Cancer Gene Ther 2020;27:528–38. 31822814 10.1038/s41417-019-0155-1

[2] Kirtane K , Elmariah H, Chung CH, Abate-Daga D. Adoptive cellular therapy in solid tumor malignancies: review of the literature and challenges ahead. J Immunother Cancer 2021;9:e002723. 34301811 10.1136/jitc-2021-002723 PMC8311333

[3] Myers JA , Miller JS. Exploring the NK cell platform for cancer immunotherapy. Nat Rev Clin Oncol 2021;18:85–100. 32934330 10.1038/s41571-020-0426-7 PMC8316981

[4] Hou B , Tang Y, Li W, Zeng Q, Chang D. Efficiency of CAR-T therapy for treatment of solid tumor in clinical trials: a meta-analysis. Dis Markers 2019;2019:3425291. 30886654 10.1155/2019/3425291 PMC6388318

[5] Dana H , Chalbatani GM, Jalali SA, Mirzaei HR, Grupp SA, Suarez ER, . CAR-T cells: early successes in blood cancer and challenges in solid tumors. Acta Pharm Sin B 2021;11:1129–47. 34094824 10.1016/j.apsb.2020.10.020 PMC8144892

[6] Vaupel P , Kallinowski F, Okunieff P. Blood flow, oxygen and nutrient supply, and metabolic microenvironment of human tumors: a review. Cancer Res 1989;49:6449–65. 2684393

[7] Stine ZE , Walton ZE, Altman BJ, Hsieh AL, Dang CV. MYC, metabolism, and cancer. Cancer Discov 2015;5:1024–39. 26382145 10.1158/2159-8290.CD-15-0507 PMC4592441

[8] Pillai SR , Damaghi M, Marunaka Y, Spugnini EP, Fais S, Gillies RJ. Causes, consequences, and therapy of tumors acidosis. Cancer Metastasis Rev 2019;38:205–22. 30911978 10.1007/s10555-019-09792-7 PMC6625890

[9] Helmlinger G , Sckell A, Dellian M, Forbes NS, Jain RK. Acid production in glycolysis-impaired tumors provides new insights into tumor metabolism. Clin Cancer Res 2002;8:1284–91. 11948144

[10] Chiche J , Ilc K, Laferrière J, Trottier E, Dayan F, Mazure NM, . Hypoxia-inducible carbonic anhydrase IX and XII promote tumor cell growth by counteracting acidosis through the regulation of the intracellular pH. Cancer Res 2009;69:358–68. 19118021 10.1158/0008-5472.CAN-08-2470

[11] Ullah MS , Davies AJ, Halestrap AP. The plasma membrane lactate transporter MCT4, but not MCT1, is up-regulated by hypoxia through a HIF-1alpha-dependent mechanism. J Biol Chem 2006;281:9030–7. 16452478 10.1074/jbc.M511397200

[12] Huber V , Camisaschi C, Berzi A, Ferro S, Lugini L, Triulzi T, . Cancer acidity: an ultimate frontier of tumor immune escape and a novel target of immunomodulation. Semin Cancer Biol 2017;43:74–89. 28267587 10.1016/j.semcancer.2017.03.001

[13] Fischer K , Hoffmann P, Voelkl S, Meidenbauer N, Ammer J, Edinger M, . Inhibitory effect of tumor cell–derived lactic acid on human T cells. Blood 2007;109:3812–9. 17255361 10.1182/blood-2006-07-035972

[14] Pötzl J , Roser D, Bankel L, Hömberg N, Geishauser A, Brenner CD, . Reversal of tumor acidosis by systemic buffering reactivates NK cells to express IFN-γ and induces NK cell-dependent lymphoma control without other immunotherapies. Int J Cancer 2017;140:2125–33. 28195314 10.1002/ijc.30646

[15] Pilon-Thomas S , Kodumudi KN, El-Kenawi AE, Russell S, Weber AM, Luddy K, . Neutralization of tumor acidity improves antitumor responses to immunotherapy. Cancer Res 2016;76:1381–90. 26719539 10.1158/0008-5472.CAN-15-1743 PMC4829106

[16] Calcinotto A , Filipazzi P, Grioni M, Iero M, De Milito A, Ricupito A, . Modulation of microenvironment acidity reverses anergy in human and murine tumor-infiltrating T lymphocytes. Cancer Res 2012;72:2746–56. 22593198 10.1158/0008-5472.CAN-11-1272

[17] Qin B-D , Jiao X-D, Zhou X-C, Shi B, Wang J, Liu K, . Effects of concomitant proton pump inhibitor use on immune checkpoint inhibitor efficacy among patients with advanced cancer. Oncoimmunology 2021;10:1929727. 34350061 10.1080/2162402X.2021.1929727 PMC8296970

[18] Beer S , Zetterberg A, Ihrie RA, McTaggart RA, Yang Q, Bradon N, . Developmental context determines latency of MYC-induced tumorigenesis. PLoS Biol 2004;2:e332. 15455033 10.1371/journal.pbio.0020332 PMC519000

[19] Walton ZE , Patel CH, Brooks RC, Yu Y, Ibrahim-Hashim A, Riddle M, . Acid suspends the circadian clock in hypoxia through inhibition of mTOR. Cell 2018;174:72–87. 29861175 10.1016/j.cell.2018.05.009 PMC6398937

[20] Gong JH , Maki G, Klingemann HG. Characterization of a human cell line (NK-92) with phenotypical and functional characteristics of activated natural killer cells. Leukemia 1994;8:652–8. 8152260

[21] Tam YK , Maki G, Miyagawa B, Hennemann B, Tonn T, Klingemann HG. Characterization of genetically altered, interleukin 2-independent natural killer cell lines suitable for adoptive cellular immunotherapy. Hum Gene Ther 1999;10:1359–73. 10365666 10.1089/10430349950018030

[22] Li J , Zhao W, Akbani R, Liu W, Ju Z, Ling S, . Characterization of human cancer cell lines by reverse-phase protein arrays. Cancer Cell 2017;31:225–39. 28196595 10.1016/j.ccell.2017.01.005 PMC5501076

[23] Michl J , Park KC, Swietach P. Evidence-based guidelines for controlling pH in mammalian live-cell culture systems. Commun Biol 2019;2:144. 31044169 10.1038/s42003-019-0393-7 PMC6486606

[24] Urano J , Comiso MJ, Guo L, Aspuria P-J, Deniskin R, Tabancay AP Jr, . Identification of novel single amino acid changes that result in hyperactivation of the unique GTPase, Rheb, in fission yeast. Mol Microbiol 2005;58:1074–86. 16262791 10.1111/j.1365-2958.2005.04877.x

[25] Webb BA , White KA, Grillo-Hill BK, Schönichen A, Choi C, Barber DL. A histidine cluster in the cytoplasmic domain of the Na-H exchanger NHE1 confers pH-sensitive phospholipid binding and regulates transporter activity. J Biol Chem 2016;291:24096–104. 27650500 10.1074/jbc.M116.736215 PMC5104935

[26] Jiang W , Hua R, Wei M, Li C, Qiu Z, Yang X, . An optimized method for high-titer lentivirus preparations without ultracentrifugation. Sci Rep 2015;5:13875. 26348152 10.1038/srep13875 PMC4562269

[27] Xiang Y , Stine ZE, Xia J, Lu Y, O'Connor RS, Altman BJ, . Targeted inhibition of tumor-specific glutaminase diminishes cell-autonomous tumorigenesis. J Clin Invest 2015;125:2293–306. 25915584 10.1172/JCI75836 PMC4497742

[28] Kim GG , Donnenberg VS, Donnenberg AD, Gooding W, Whiteside TL. A novel multiparametric flow cytometry-based cytotoxicity assay simultaneously immunophenotypes effector cells: comparisons to a 4 h 51Cr-release assay. J Immunol Methods 2007;325:51–66. 17617419 10.1016/j.jim.2007.05.013 PMC2040258

[29] Burshtyn DN , Davidson C. Natural killer cell conjugate assay using two-color flow cytometry. Methods Mol Biol 2010;612:89–96. 20033636 10.1007/978-1-60761-362-6_7

[30] Lorenzo-Herrero S , Sordo-Bahamonde C, Gonzalez S, López-Soto A. CD107a degranulation assay to evaluate immune cell antitumor activity. Methods Mol Biol 2019;1884:119–30. 30465198 10.1007/978-1-4939-8885-3_7

[31] Wieder ED , Hang H, Fox MH. Measurement of intracellular pH using flow cytometry with carboxy-SNARF-1. Cytometry 1993;14:916–21. 8287734 10.1002/cyto.990140810

[32] Dong Y , Gao Y, Ilie A, Kim D, Boucher A, Li B, . Structure and mechanism of the human NHE1-CHP1 complex. Nat Commun 2021;12:3474. 34108458 10.1038/s41467-021-23496-z PMC8190280

[33] Hsu H-T , Carisey AF, Orange JS. Measurement of lytic granule convergence after formation of an NK cell immunological synapse. Methods Mol Biol 2017;1584:497–515. 28255722 10.1007/978-1-4939-6881-7_31 PMC5861262

[34] Menon S , Yecies JL, Zhang HH, Howell JJ, Nicholatos J, Harputlugil E, . Chronic activation of mTOR complex 1 is sufficient to cause hepatocellular carcinoma in mice. Sci Signal 2012;5:ra24. 22457330 10.1126/scisignal.2002739 PMC3743103

[35] Klingemann H , Boissel L, Toneguzzo F. Natural killer cells for immunotherapy – advantages of the NK-92 cell line over blood NK cells. Front Immunol 2016;7:91. 27014270 10.3389/fimmu.2016.00091 PMC4789404

[36] Fabian KP , Hodge JW. The emerging role of off-the-shelf engineered natural killer cells in targeted cancer immunotherapy. Mol Ther Oncolytics 2021;23:266–76. 34761106 10.1016/j.omto.2021.10.001 PMC8560822

[37] Suck G , Odendahl M, Nowakowska P, Seidl C, Wels WS, Klingemann HG, . NK-92: an ‘off-the-shelf therapeutic’ for adoptive natural killer cell-based cancer immunotherapy. Cancer Immunol Immunother 2016;65:485–92. 26559813 10.1007/s00262-015-1761-x PMC11029582

[38] Smalley KSM , Xiao M, Villanueva J, Nguyen TK, Flaherty KT, Letrero R, . CRAF inhibition induces apoptosis in melanoma cells with non-V600E BRAF mutations. Oncogene 2009;28:85–94. 18794803 10.1038/onc.2008.362 PMC2898184

[39] Donnelly RP , Loftus RM, Keating SE, Liou KT, Biron CA, Gardiner CM, . mTORC1-dependent metabolic reprogramming is a prerequisite for NK cell effector function. J Immunol 2014;193:4477–84. 25261477 10.4049/jimmunol.1401558 PMC4201970

[40] Wang F , Meng M, Mo B, Yang Y, Ji Y, Huang P, . Crosstalks between mTORC1 and mTORC2 variagate cytokine signaling to control NK maturation and effector function. Nat Commun 2018;9:4874. 30451838 10.1038/s41467-018-07277-9 PMC6242843

[41] Veliça P , Zech M, Henson S, Holler A, Manzo T, Pike R, . Genetic regulation of fate decisions in therapeutic T cells to enhance tumor protection and memory formation. Cancer Res 2015;75:2641–52. 25904681 10.1158/0008-5472.CAN-14-3283

[42] Pollizzi KN , Patel CH, Sun I-H, Oh M-H, Waickman AT, Wen J, . mTORC1 and mTORC2 selectively regulate CD8^+^ T cell differentiation. J Clin Invest 2015;125:2090–108. 25893604 10.1172/JCI77746 PMC4463194

[43] Ditte P , Dequiedt F, Svastova E, Hulikova A, Ohradanova-Repic A, Zatovicova M, . Phosphorylation of carbonic anhydrase IX controls its ability to mediate extracellular acidification in hypoxic tumors. Cancer Res 2011;71:7558–67. 22037869 10.1158/0008-5472.CAN-11-2520

[44] Karbowniczek M , Cash T, Cheung M, Robertson GP, Astrinidis A, Henske EP. Regulation of B-raf kinase activity by tuberin and Rheb is mammalian target of rapamycin (mTOR)-independent. J Biol Chem 2004;279:29930–7. 15150271 10.1074/jbc.M402591200

[45] Busco G , Cardone RA, Greco MR, Bellizzi A, Colella M, Antelmi E, . NHE1 promotes invadopodial ECM proteolysis through acidification of the peri-invadopodial space. FASEB J 2010;24:3903–15. 20547664 10.1096/fj.09-149518

[46] Fafournoux P , Noël J, Pouysségur J. Evidence that Na^+^/H^+^ exchanger isoforms NHE1 and NHE3 exist as stable dimers in membranes with a high degree of specificity for homodimers. J Biol Chem 1994;269:2589–96. 8300588

[47] Chen X , Allan DSJ, Krzewski K, Ge B, Kopcow H, Strominger JL. CD28-stimulated ERK2 phosphorylation is required for polarization of the microtubule organizing center and granules in YTS NK cells. Proc Natl Acad Sci U S A 2006;103:10346–51. 16801532 10.1073/pnas.0604236103 PMC1502460

[48] Hendus-Altenburger R , Pedraz-Cuesta E, Olesen CW, Papaleo E, Schnell JA, Hopper JTS, . The human Na(+)/H(+) exchanger 1 is a membrane scaffold protein for extracellular signal-regulated kinase 2. BMC Biol 2016;14:31. 27083547 10.1186/s12915-016-0252-7 PMC4833948

[49] Loftus RM , Assmann N, Kedia-Mehta N, O'Brien KL, Garcia A, Gillespie C, . Amino acid-dependent cMyc expression is essential for NK cell metabolic and functional responses in mice. Nat Commun 2018;9:2341. 29904050 10.1038/s41467-018-04719-2 PMC6002377

[50] Van Riggelen J , Yetil A, Felsher DW. MYC as a regulator of ribosome biogenesis and protein synthesis. Nat Rev Cancer 2010;10:301–9. 20332779 10.1038/nrc2819

[51] Gallagher FA , Kettunen MI, Day SE, Hu D-E, Ardenkjaer-Larsen JH, Zandt RI, . Magnetic resonance imaging of pH in vivo using hyperpolarized 13C-labelled bicarbonate. Nature 2008;453:940–3. 18509335 10.1038/nature07017

[52] Chao M , Wu H, Jin K, Li B, Wu J, Zhang G, . A nonrandomized cohort and a randomized study of local control of large hepatocarcinoma by targeting intratumoral lactic acidosis. Elife 2016;5:e15691. 27481188 10.7554/eLife.15691 PMC4970867

[53] Rajkumar P , Pluznick JL. Acid-base regulation in the renal proximal tubules: using novel pH sensors to maintain homeostasis. Am J Physiol Renal Physiol 2018;315:F1187–90. 30066586 10.1152/ajprenal.00185.2018 PMC6293293

[54] Oppersma E , Doorduin J, van der Hoeven JG, Veltink PH, van Hees HWH, Heunks LMA. The effect of metabolic alkalosis on the ventilatory response in healthy subjects. Respir Physiol Neurobiol 2018;249:47–53. 29307724 10.1016/j.resp.2018.01.002

[55] Justus CR , Dong L, Yang LV. Acidic tumor microenvironment and pH-sensing G protein-coupled receptors. Front Physiol 2013;4:354. 24367336 10.3389/fphys.2013.00354 PMC3851830

[56] Fliegel L . Structural and functional changes in the Na^+^/H^+^ exchanger isoform 1, induced by Erk1/2 phosphorylation. Int J Mol Sci 2019;20:2378. 31091671 10.3390/ijms20102378 PMC6566726

[57] Li Y , Li D, Liu Y, Wang S, Sun M, Zhang Z, . The positive feedback loop of NHE1-ERK phosphorylation mediated by BRAF^V600E^ mutation contributes to tumorigenesis and development of glioblastoma. Biochem Biophys Res Commun 2022;588:1–7. 34933181 10.1016/j.bbrc.2021.11.104

[58] Ben-Shmuel A , Sabag B, Biber G, Barda-Saad M. The role of the cytoskeleton in regulating the natural killer cell immune response in health and disease: from signaling dynamics to function. Front Cell Dev Biol 2021;9:609532. 33598461 10.3389/fcell.2021.609532 PMC7882700

[59] Denker SP , Huang DC, Orlowski J, Furthmayr H, Barber DL. Direct Binding of the Na–H exchanger NHE1 to erm proteins regulates the cortical cytoskeleton and cell shape independently of H(+) translocation. Mol Cell 2000;6:1425–36. 11163215 10.1016/s1097-2765(00)00139-8

[60] Denker SP , Barber DL. Cell migration requires both ion translocation and cytoskeletal anchoring by the Na-H exchanger NHE1. J Cell Biol 2002;159:1087–96. 12486114 10.1083/jcb.200208050 PMC2173980

[61] Persi E , Duran-Frigola M, Damaghi M, Roush WR, Aloy P, Cleveland JL, . Systems analysis of intracellular pH vulnerabilities for cancer therapy. Nat Commun 2018;9:2997. 30065243 10.1038/s41467-018-05261-x PMC6068141

[62] Man CH , Mercier FE, Liu N, Dong W, Stephanopoulos G, Jiang L, . Proton export alkalinizes intracellular pH and reprograms carbon metabolism to drive normal and malignant cell growth. Blood 2022;139:502–22. 34610101 10.1182/blood.2021011563 PMC8796654

[63] Di Stasi A , De Angelis B, Rooney CM, Zhang L, Mahendravada A, Foster AE, . T lymphocytes coexpressing CCR4 and a chimeric antigen receptor targeting CD30 have improved homing and antitumor activity in a hodgkin tumor model. Blood 2009;113:6392–402. 19377047 10.1182/blood-2009-03-209650 PMC2710932

[64] Huang X , Guo H, Kang J, Choi S, Zhou TC, Tammana S, . Sleeping Beauty transposon-mediated engineering of human primary T cells for therapy of CD19+ lymphoid malignancies. Mol Ther 2008;16:580–9. 18227839 10.1038/sj.mt.6300404 PMC4539139

[65] Uzhachenko RV , Shanker A. CD8^+^ T lymphocyte and NK cell network: circuitry in the cytotoxic domain of immunity. Front Immunol 2019;10:1906. 31456803 10.3389/fimmu.2019.01906 PMC6700470

