# Supplementary material for: Na+/H+-exchanger 1 Enhances Antitumor Activity of Engineered NK-92 Natural Killer Cells
Source: Cancer Res Commun. 2022 Aug 22;2(8):842–56. doi: 10.1158/2767-9764.CRC-22-0270 (PMC9648415; doi:10.1158/2767-9764.CRC-22-0270)
Supplement: Supplementary Figures 1-5, Tables 1-2 — Supplementary Fig. S1. CA9 fails to enhance the cytolytic activity of NK-92 cells in vitro. Supplementary Fig. S2. NHE1 enhances in vitro cytotoxicity of NK-92 cells without affecting proliferation or survival. Supplementary Fig. S3. Representative three-dimensional (3D) reconstruction of confocal microscopic images of antibody-activated NK-92. Supplementary Fig. S4. NHE1 increases the protein expression of c-Myc in NK-92 cells. Supplementary Fig. S5. NHE1 enhances in vitro cytotoxicity of NK-92MI. Supplementary Table S1. Nucleotide sequence of codon-optimized, constitutively active human NHE1 cDNA. Supplementary Table S2. Differentially expressed genes between Na+/H+-exchanger 1 (NHE1)-expressing and empty vector NK-92 cells, ranked by log2(fold change). [file crc-22-0270-s01.docx]

# Supplementary data


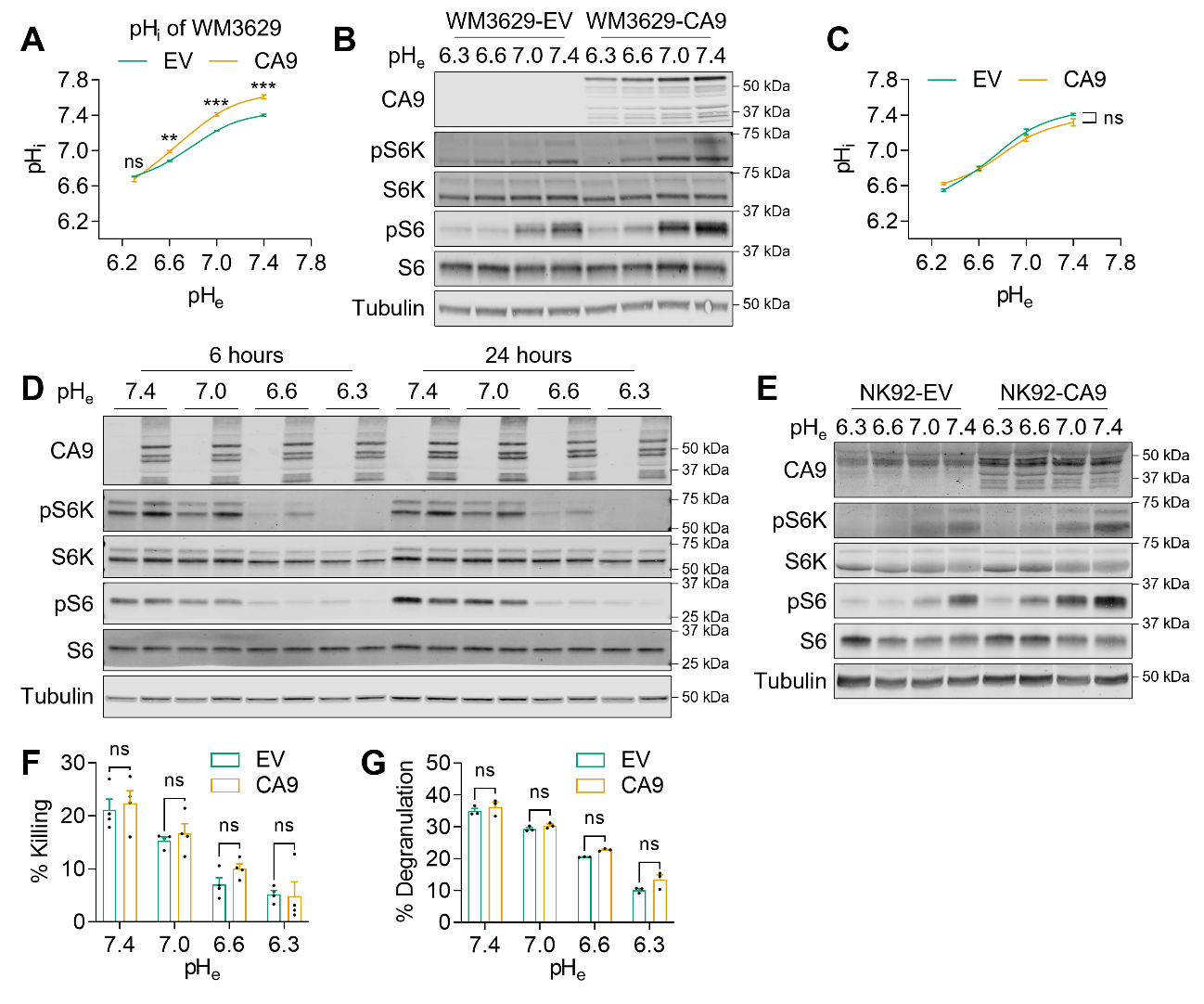


## Supplementary Fig. S1. CA9 fails to enhance the cytolytic activity of NK-92 cells in vitro.

(**A**) Baseline intracellular pH (pH_i_) of WM3629 human melanoma cells expressing CA9 or empty vector (EV) control in pH-controlled media (defining extracellular pH, or pH_e_) (N = 3 wells, two-way ANOVA with multiple comparisons). (**B**) Representative immunoblots of mTORC1 targets phospho-p70 S6 kinase (pS6K) and phospho-S6 ribosomal protein (pS6) in WM3629 cells expressing CA9 or EV treated with pH-controlled media (defining extracellular pH, or pH_e_) for 6 hours. At the exposure shown, endogenous CA9 is barely visible due to low expression. (**C**) Baseline pH_i_ of NK-92 cells expressing CA9 or EV control in pH-controlled media (N = 3 wells, two-way ANOVA). (**D**) and (**E**) Representative immunoblots of mTORC1 targets pS6K and pS6 in NK-92 cells expressing CA9 or EV treated with pH-controlled media for 6 or 24 hours (**D**) and 6 hours (**E**). (**F**) In vitro cytotoxicity of NK-92 cells expressing CA9 or EV against WM3629 cells in pH-controlled media (N = 4 wells, unpaired t-test). (**G**) Degranulation of NK-92 cells expressing CA9 or EV towards K562 cells in pH-controlled media (N = 3 wells, unpaired t-test). ns not significant, ** p < 0.01, *** p < 0.001.


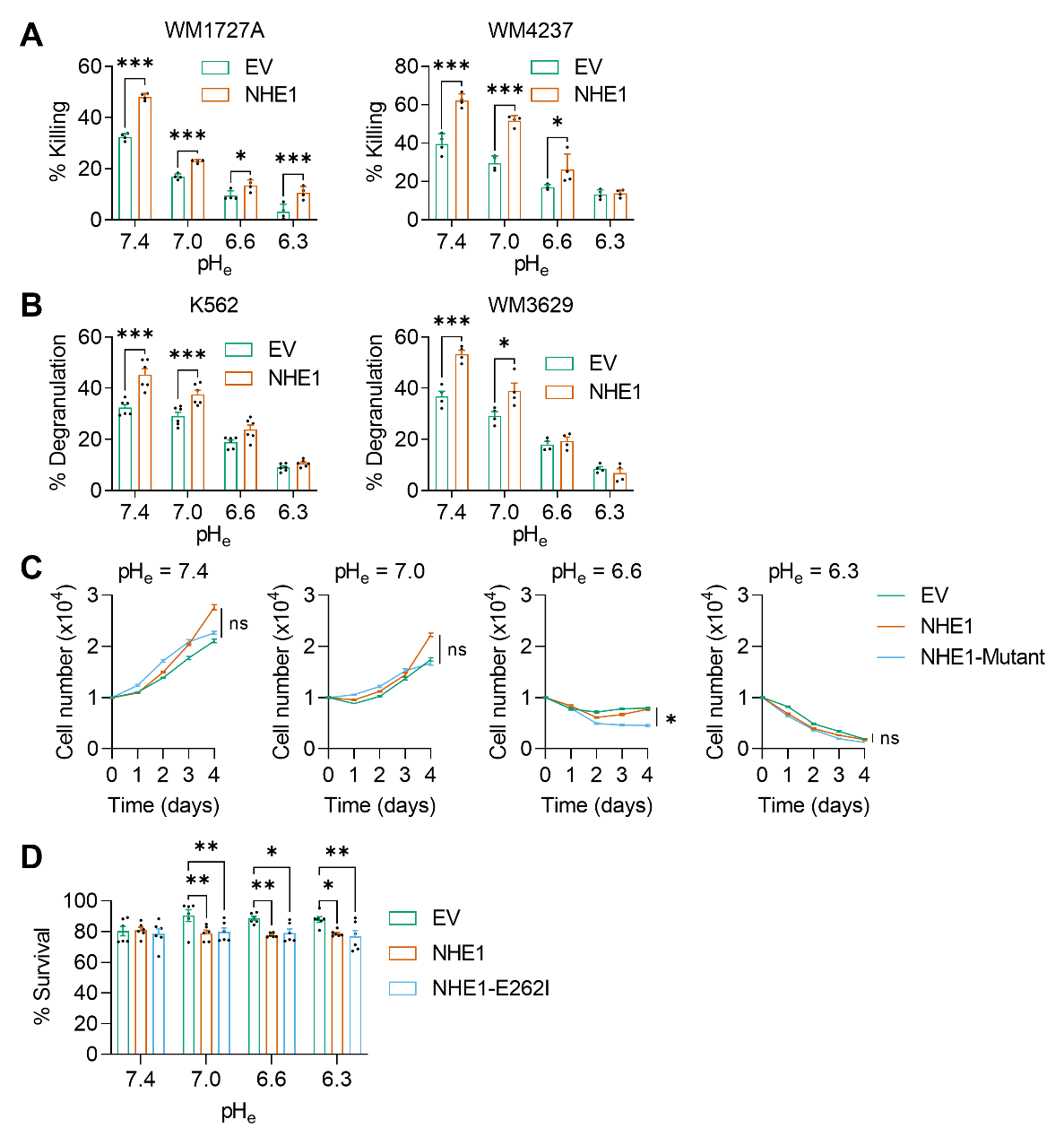


## Supplementary Fig. S2. NHE1 enhances in vitro cytotoxicity of NK-92 cells without affecting proliferation or survival.

(**A**) In vitro cytotoxicity of NK-92 cells expressing constitutively active NHE1 (referred to as NHE1) or empty vector (EV) control against human melanoma cell lines WM1727A and WM4237 in pH-controlled media (defining extracellular pH, or pH_e_) (N = 4, unpaired t-test). (**B**) Degranulation of NK-92 cells expressing NHE1 or EV towards K562 cells and WM3629 cells in pH-controlled media (N = 6 wells and pooled from two independent experiments for K562, N = 4 wells for WM3629, two-way ANOVA with multiple comparisons). (**C**) Proliferation of NK-92 cells expressing NHE1, inactive NHE1 (referred to as NHE1-E262I), or EV over time in pH-controlled media (N = 6, two-way ANOVA). (**D**) Survival of NK-92 cells expressing NHE1, NHE1-E262I, or EV after cytotoxicity with WM3629 human melanoma cells (N = 6, two-way ANOVA with multiple comparisons). * p < 0.05, ** p < 0.01, *** p < 0.001, ns not significant.


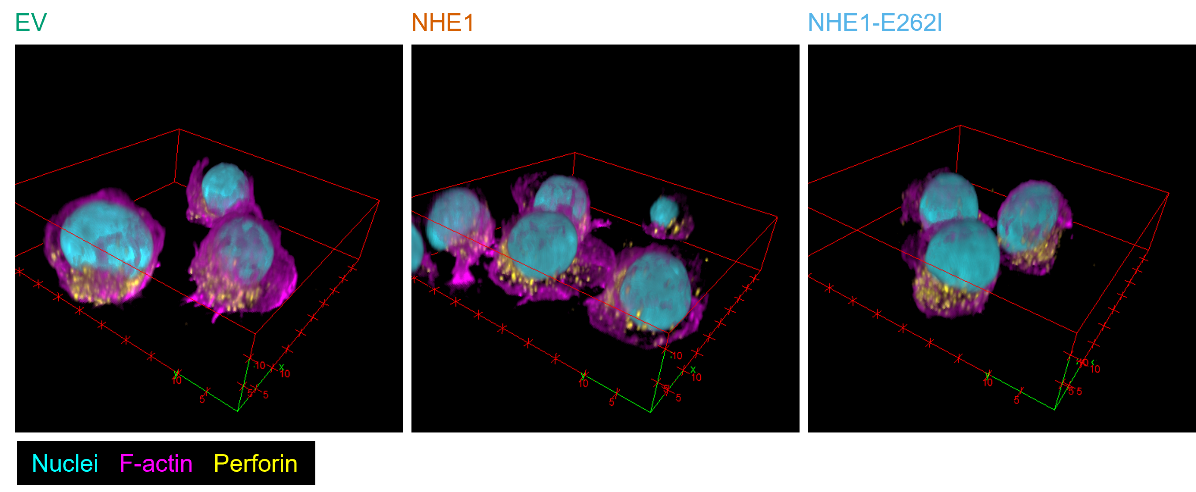


## Supplementary Fig. S3. Representative three-dimensional (3D) reconstruction of confocal microscopic images of antibody-activated NK-92.

NK-92 cells expressing NHE1, NHE1-E262I, or EV, with fluorescent labeling of nuclei (DAPI, cyan) and F-actin (phalloidin, magenta), and immunofluorescence of perforin (yellow).


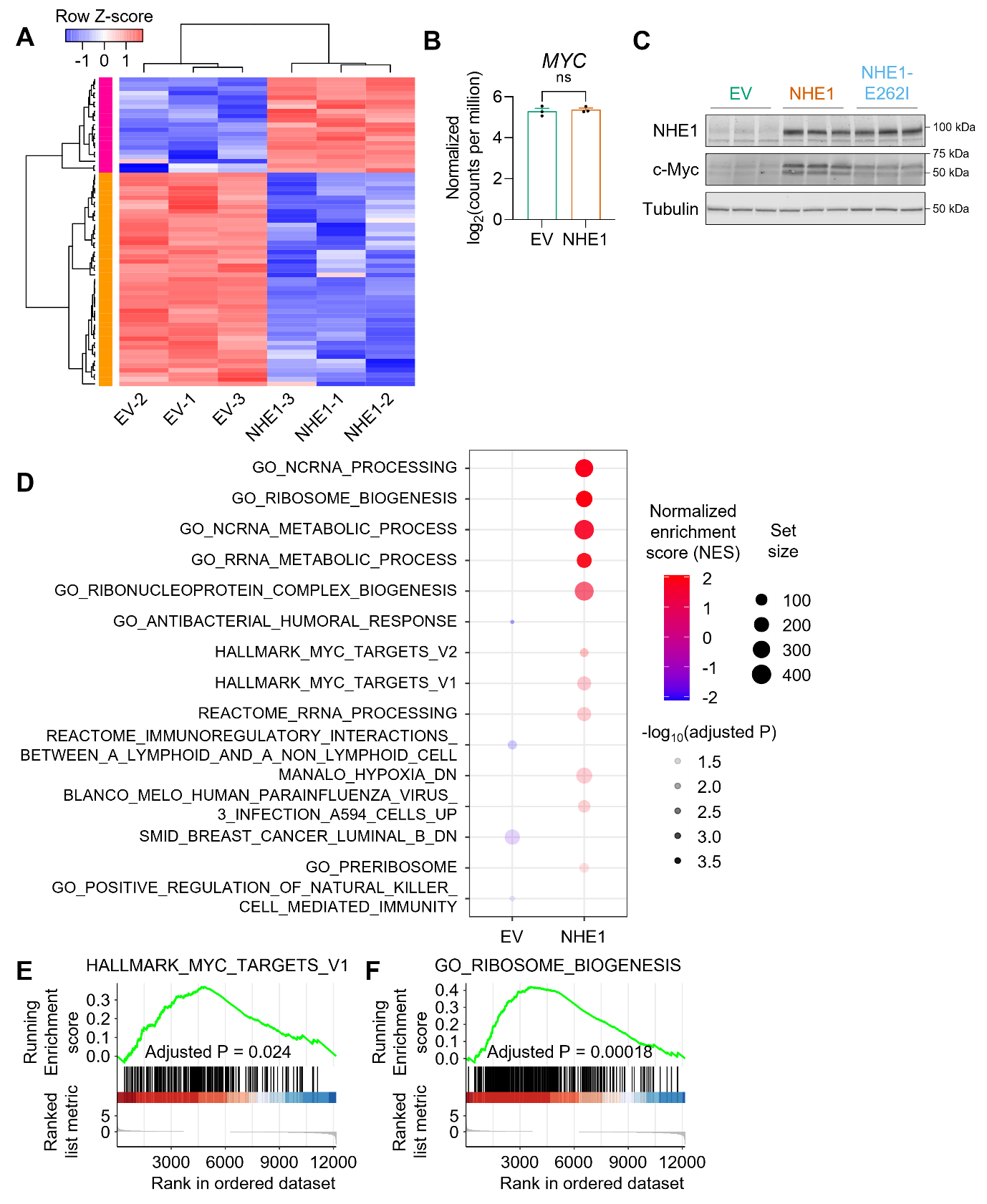


## Supplementary Fig. S4. NHE1 increases the protein expression of c-Myc in NK-92 cells.

(**A**) Normalized, log_2_-transformed counts per million (CPM) of *MYC* transcript in NK-92 cells expressing EV or NHE1 measured by QuantSeq 3' mRNA-sequencing (N = 3 samples, unpaired t-test). (**B**) Representative immunoblots of c-Myc in NK-92 cells expressing NHE1, NHE1-E262I, or EV at basal conditions. (**C**) and (**D**) Enrichment plots of the Myc target (left) and ribosomal biogenesis (right) gene sets enriched in NHE1-expressing NK-92 cells compared with empty vector cells. ns not significant. (**E**) Heatmap of all differentially expressed genes with log_2_(fold change) > 1 and p < 0.05 (N = 3 samples). (**F**) Enrichment of gene sets representing potential targets of regulation by transcription factors or microRNAs (curated in category C3 of the MSigDB) in genes upregulated in NHE1 (upper) or EV (lower) cells. (**G**) Enriched gene sets in NHE1- or EV-expressing NK-92 cells.


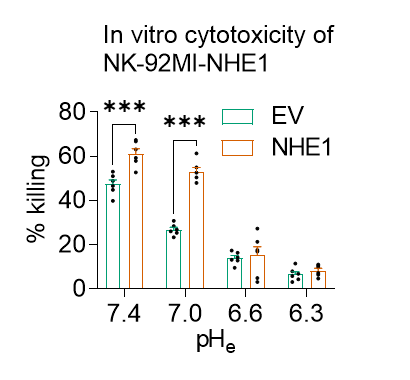


## Supplementary Fig. S5. NHE1 enhances in vitro cytotoxicity of NK-92MI.

In vitro cytotoxicity of NK-92MI cells expressing NHE1 or EV against WM3629 cells in pH-controlled media (defining extracellular pH, or pH_e_) (N = 6, unpaired t-test). *** p < 0.001.

## Supplementary Table S1. Nucleotide sequence of codon-optimized, constitutively active human NHE1 cDNA.

Highlighted nucleotides denote the arginine residues mutated from the proton-sensing histidine residues in the wild-type NHE1 cDNA to confer constitutive activation.

| ATGGTGCTGAGGAGTGGTATCTGCGGCCTGTCCCCCCATAGGATATTTCCAAGTTTGCTTGTAGTTGTAGCTCTCGTCGGATTGCTCCCTGTTCTGCGCTCTCACGGACTGCAACTGTCTCCGACTGCTTCCACTATTCGGTCATCTGAGCCACCGCGCGAGAGGAGCATCGGGGATGTTACTACAGCACCACCAGAGGTCACCCCCGAGTCACGACCAGTGAACCACTCCGTCACTGATCATGGGATGAAGCCGCGGAAGGCTTTCCCCGTGCTCGGGATTGATTACACGCATGTACGGACACCTTTTGAAATCTCACTCTGGATCCTGTTGGCGTGTCTCATGAAAATCGGGTTTCATGTAATACCGACGATTTCTTCCATCGTGCCAGAGTCTTGTCTCCTCATTGTGGTCGGTCTCCTCGTTGGCGGTCTCATCAAGGGAGTTGGCGAGACACCGCCGTTTTTGCAATCAGATGTATTCTTTTTGTTTCTTCTGCCCCCAATAATTCTTGATGCAGGGTATTTCTTGCCGCTCAGACAGTTTACTGAGAACCTTGGGACTATACTTATATTCGCGGTAGTAGGAACCCTCTGGAACGCCTTTTTCCTGGGAGGGTTGATGTACGCTGTATGTCTCGTCGGTGGAGAGCAAATTAACAATATTGGTCTGTTGGACAATCTTTTGTTCGGCTCCATAATCAGCGCTGTCGATCCAGTCGCCGTGCTCGCTGTATTCGAGGAAATCCACATCAACGAACTTCTTCATATACTCGTTTTCGGTGAAAGTCTTCTCAATGATGCCGTGACTGTAGTTCTTTACCATCTCTTCGAAGAGTTCGCCAACTATGAGCACGTTGGAATAGTCGATATTTTCCTTGGGTTTCTCTCTTTCTTCGTCGTTGCCCTCGGAGGAGTCTTGGTAGGCGTCGTCTACGGCGTCATAGCAGCCTTTACTTCTAGGTTTACGTCTCACATACGCGTGATTGAGCCGTTGTTTGTTTTTCTGTATTCCTATATGGCCTATTTGAGTGCCGAGCTTTTTCATCTTAGCGGTATAATGGCCCTTATCGCGTCTGGGGTTGTCATGCGCCCATATGTCGAGGCGAATATAAGTCACAAATCCCATACCACGATTAAATATTTCCTCAAAATGTGGTCAAGCGTTTCAGAAACCCTTATATTCATATTCCTGGGAGTCAGCACAGTAGCGGGCTCCCATCACTGGAACTGGACATTCGTAATATCTACGTTGCTCTTTTGCCTGATAGCCAGAGTTCTGGGCGTGCTCGGACTGACTTGGTTTATTAACAAATTCAGAATTGTTAAACTGACGCCTAAAGACCAGTTCATCATAGCATATGGAGGTTTGCGCGGGGCAATCGCATTCAGTCTGGGGTATCTCCTCGACAAGAAGCACTTCCCCATGTGCGATCTGTTTTTGACCGCGATCATCACAGTCATATTTTTTACGGTTTTTGTACAGGGGATGACCATCAGGCCACTCGTTGATCTTTTGGCGGTCAAAAAAAAACAAGAGACGAAACGAAGTATAAATGAAGAGATACATACTCAGTTCTTGGACCACTTGCTGACCGGGATAGAGGACATTTGTGGCCGCTATGGCAGGCGACGATGGAAGGATAAACTGAATCGGTTTAACAAAAAATATGTGAAAAAATGCTTGATCGCCGGGGAACGGTCTAAAGAACCACAGCTTATAGCCTTCTATCATAAAATGGAGATGAAGCAGGCGATAGAGCTGGTGGAATCCGGAGGAATGGGAAAGATACCCAGCGCTGTCTCAACCGTGTCTATGCAAAATATCCATCCGAAGTCCCTTCCATCTGAGCGAATCCTGCCCGCCCTCAGCAAGGACAAAGAGGAGGAGATTCGGAAAATTCTGAGGAATAACTTGCAGAAGACTAGACAGCGCCTCAGATCCTATAACCGACACACCCTGGTGGCCGACCCCTATGAGGAAGCCTGGAACCAGATGTTGCTTCGACGGCAAAAAGCTCGACAATTGGAGCAAAAGATCAATAACTATCTCACCGTCCCTGCTCACAAACTTGACTCTCCCACTATGTCTCGAGCCAGGATAGGATCTGACCCCCTGGCGTACGAGCCAAAAGAGGATTTGCCTGTCATTACGATAGATCCGGCCTCCCCGCAGTCTCCCGAGTCCGTAGACCTGGTTAACGAGGAACTTAAGGGCAAAGTTCTGGGCCTTAGTCGGGATCCGGCAAAGGTTGCTGAGGAGGACGAAGATGATGATGGGGGTATTATGATGAGGTCAAAAGAAACAAGTTCCCCCGGTACGGACGATGTATTCACGCCGGCGCCTTCTGACTCCCCAAGCTCTCAACGCATACAGCGGTGCCTGAGTGACCCGGGGCCCCATCCGGAGCCGGGTGAAGGGGAGCCGTTTTTTCCTAAAGGCCAATAG |
| --- |

## Supplementary Table S2. Differentially expressed genes between Na^+^/H^+^-exchanger 1 (NHE1)-expressing and empty vector NK-92 cells, ranked by log_2_(fold change).

| Gene symbol | log2(fold change) | Average log expression | t | P value | Adjusted P value |
| --- | --- | --- | --- | --- | --- |
| *SCO2* | 5.103573 | -0.54501915 | 4.9902 | 0.00041 | 0.02475 |
| *ITGB1P1* | 4.645597145 | 0.484581501 | 5.2806 | 0.00026 | 0.01875 |
| *TRIM9* | 3.169068388 | 0.775067549 | 6.735 | 3.23E-05 | 0.00538 |
| *SVIL* | 2.499327608 | 0.405048831 | 5.313 | 0.00025 | 0.01827 |
| *KDM5B* | 2.34695473 | 3.309948129 | 8.4944 | 3.70E-06 | 0.00132 |
| *S100A8* | 1.736219927 | 7.360837922 | 23.245 | 1.07E-10 | 6.52E-07 |
| *NTRK2* | 1.563991339 | 2.712950259 | 6.8571 | 2.75E-05 | 0.00471 |
| *SMAD3* | 1.551572451 | 3.673007692 | 8.9738 | 2.17E-06 | 0.00101 |
| *MYO1E* | 1.50557722 | 3.191612883 | 8.6614 | 3.06E-06 | 0.00116 |
| *FMNL2* | 1.500968015 | 1.470190094 | 5.1468 | 0.00032 | 0.02152 |
| *GABRB3* | 1.419259878 | 4.48649066 | 9.8335 | 8.80E-07 | 0.00063 |
| *RBM41* | 1.367224103 | 1.631172352 | 4.5659 | 0.00081 | 0.03602 |
| *P2RX5* | 1.343992755 | 2.919964421 | 5.9879 | 9.11E-05 | 0.01045 |
| *PDGFRL* | 1.326987156 | 1.624479413 | 4.2176 | 0.00144 | 0.04843 |
| *ANO3* | 1.291170467 | 2.188159282 | 4.288 | 0.00128 | 0.04678 |
| *INHBA* | 1.283301556 | 2.644965289 | 5.6657 | 0.00015 | 0.01363 |
| *TMEM217* | 1.136889448 | 1.591037007 | 4.2394 | 0.00139 | 0.04822 |
| *ZSCAN5A* | 1.088095058 | 2.106319022 | 4.4957 | 0.00091 | 0.03803 |
| *DENND1A* | 1.041340238 | 2.245006385 | 4.2063 | 0.00147 | 0.04843 |
| *SLAMF8* | 1.031124736 | 3.418922056 | 5.7039 | 0.00014 | 0.01339 |
| *GZMH* | -1.004760947 | 4.827554777 | -8.663 | 3.06E-06 | 0.00116 |
| *OSBPL5* | -1.011549762 | 2.966947793 | -5.5213 | 0.00018 | 0.01491 |
| *TIMP1* | -1.014456484 | 4.939141088 | -7.1161 | 1.96E-05 | 0.00382 |
| *CTLA4* | -1.020656956 | 4.479071999 | -9.1913 | 1.72E-06 | 0.00087 |
| *SEPTIN8* | -1.029990324 | 3.645980533 | -5.5743 | 0.00017 | 0.01457 |
| *IL27RA* | -1.051969557 | 4.784294875 | -9.5035 | 1.23E-06 | 0.00072 |
| *SERPINB6* | -1.058408586 | 2.899745041 | -5.5187 | 0.00018 | 0.01491 |
| *RUNX1T1* | -1.10608903 | 4.891450744 | -7.7686 | 8.67E-06 | 0.00245 |
| *GZMB* | -1.115769822 | 10.29985992 | -25.422 | 4.08E-11 | 4.96E-07 |
| *TET2* | -1.122959269 | 1.776029623 | -4.1829 | 0.00153 | 0.0486 |
| *TREM1* | -1.188254708 | 3.782253423 | -7.1703 | 1.83E-05 | 0.00377 |
| *SEMA4A* | -1.218369696 | 2.732994271 | -5.4097 | 0.00021 | 0.01636 |
| *LILRA2* | -1.255456626 | 2.966749373 | -4.872 | 0.00049 | 0.02775 |
| *MSANTD3-TMEFF1* | -1.279677297 | 2.443152468 | -4.8564 | 0.00051 | 0.02825 |
| *ADTRP* | -1.290842741 | 3.465986165 | -7.6557 | 9.94E-06 | 0.00252 |
| *DMKN* | -1.29132268 | 2.498078778 | -5.2083 | 0.00029 | 0.02036 |
| *CD160* | -1.397084235 | 4.336829466 | -8.0044 | 6.53E-06 | 0.00194 |
| *FCRL4* | -1.414813415 | 2.976854057 | -6.3475 | 5.48E-05 | 0.00757 |
| *CLU* | -1.449939048 | 7.120337445 | -17.013 | 3.04E-09 | 1.23E-05 |
| *LINC01881* | -1.481100897 | 1.373177843 | -4.4472 | 0.00099 | 0.03964 |
| *HACD4* | -1.489528241 | 1.759461857 | -5.2715 | 0.00026 | 0.01879 |
| *KIAA0930* | -1.527637008 | 2.025573185 | -5.1108 | 0.00034 | 0.02216 |
| *SPINK5* | -1.549607724 | 4.515102644 | -11.808 | 1.38E-07 | 0.00021 |
| *CD86* | -1.640925194 | 2.801591861 | -6.5039 | 4.42E-05 | 0.00675 |
| *LINGO1* | -1.736364002 | 1.630969384 | -5.4775 | 0.00019 | 0.01528 |
| *AC012254.2* | -1.772455825 | 1.960333864 | -4.5319 | 0.00086 | 0.03732 |
| *IL1R1* | -1.849176197 | 1.039660098 | -4.975 | 0.00042 | 0.025 |
| *ALDH1L1* | -1.922106282 | 0.658622937 | -4.7772 | 0.00058 | 0.02976 |
| *CCR7* | -2.0607137 | 2.490946293 | -8.9082 | 2.33E-06 | 0.00101 |
| *DNASE2* | -2.137496925 | 2.94845012 | -10.842 | 3.30E-07 | 0.00043 |
| *NRXN3* | -2.202915542 | 1.91386585 | -6.6192 | 3.78E-05 | 0.00604 |
| *IL18RAP* | -2.289036176 | 2.415404288 | -8.0743 | 6.01E-06 | 0.00187 |
| *IL1R2* | -2.366774096 | 0.584346302 | -5.2196 | 0.00029 | 0.02012 |
| *BTBD3* | -2.471931273 | 0.811450097 | -4.8021 | 0.00055 | 0.02914 |
| *ARAP3* | -2.526530593 | -0.420092111 | -4.2619 | 0.00134 | 0.04762 |
| *NFE2* | -2.6336511 | -0.185243961 | -4.814 | 0.00054 | 0.02914 |
| *HOMER1* | -2.816204192 | 1.869948617 | -9.7079 | 9.99E-07 | 0.00064 |
| *NAALADL2* | -2.831367255 | -0.100897893 | -4.2901 | 0.00128 | 0.04678 |
| *NCR2* | -2.838997312 | 1.111831082 | -7.4424 | 1.30E-05 | 0.00309 |
| *SPTA1* | -2.855696784 | -0.877266296 | -4.8007 | 0.00055 | 0.02914 |
| *SLPI* | -2.856383197 | -1.001697612 | -4.6074 | 0.00076 | 0.03499 |
| *ST7* | -2.942593185 | 0.217468515 | -5.9286 | 9.93E-05 | 0.01107 |
| *TLL1* | -3.311098714 | -0.909809451 | -5.4238 | 0.00021 | 0.01622 |
| *GLP1R* | -3.806179709 | 0.954122542 | -8.1236 | 5.67E-06 | 0.00181 |
| *ANGPT1* | -3.991621564 | 0.68948328 | -8.387 | 4.18E-06 | 0.00145 |
| *NR2F2* | -4.206919832 | 0.034316434 | -6.8947 | 2.62E-05 | 0.00454 |
| *AP003419.1* | -5.897449991 | -2.012793747 | -10.658 | 3.92E-07 | 0.00043 |
